# Investigating the Role of 17-Beta Estradiol in the Regulation of the Unfolded Protein Response (UPR) in Pancreatic Beta Cells

**DOI:** 10.3390/ijms25031816

**Published:** 2024-02-02

**Authors:** Monica De Paoli, Deep Shah, Alexander Zakharia, Zil Patel, Zinal Patel, Pakhi Pakhi, Geoff H. Werstuck

**Affiliations:** 1 Department of Medicine, Faculty of Health Sciences, McMaster University, 1280 Main Street W, Hamilton, ON L8S 4L8, Canada; depaolim@mcmaster.ca (M.D.P.); zilpatel2000@gmail.com (Z.P.); patelzin12@gmail.com (Z.P.);; 2 Thrombosis and Atherosclerosis Research Institute, 237 Barton Street E, Hamilton, ON L8L 2X2, Canada

**Keywords:** diabetes, 17-beta estradiol, endoplasmic reticulum stress, unfolded protein response, pancreatic beta cells

## Abstract

Diabetes mellitus is clinically defined by chronic hyperglycemia. Sex differences in the presentation and outcome of diabetes exist with premenopausal women having a reduced risk of developing diabetes, relative to men, or women after menopause. Accumulating evidence shows a protective role of estrogens, specifically 17-beta estradiol, in the maintenance of pancreatic beta cell health; however, the mechanisms underlying this protection are still unknown. To elucidate these potential mechanisms, we used a pancreatic beta cell line (BTC6) and a mouse model of hyperglycemia-induced atherosclerosis, the ApoE^−/−^:Ins2^+/Akita^ mouse, exhibiting sexual dimorphism in glucose regulation. In this study we hypothesize that 17-beta estradiol protects pancreatic beta cells by modulating the unfolded protein response (UPR) in response to endoplasmic reticulum (ER) stress. We observed that ovariectomized female and male ApoE^−/−^:Ins2^+/Akita^ mice show significantly increased expression of apoptotic UPR markers. Sham operated female and ovariectomized female ApoE^−/−^:Ins2^+/Akita^ mice supplemented with exogenous 17-beta estradiol increased the expression of adaptive UPR markers compared to non-supplemented ovariectomized female ApoE^−/−^:Ins2^+/Akita^ mice. These findings were consistent to what was observed in cultured BTC6 cells, suggesting that 17-beta estradiol may protect pancreatic beta cells by repressing the apoptotic UPR and enhancing the adaptive UPR activation in response to pancreatic ER stress.

## 1. Introduction

Diabetes mellitus is a metabolic disorder characterized by the inability of pancreatic beta cells to secrete sufficient insulin to adequately regulate blood glucose levels [1]. This condition is associated with a variety of vascular disorders including an increased risk of developing atherosclerosis, the underlying cause of cardiovascular diseases [2]. The prevalence and incidence of diabetes mellitus has been increasing worldwide due to a more sedentary lifestyle and an increased prevalence of obesity. As a result, diabetes has become a significant burden on health care and health care costs [1]. 

Sex differences in the prevalence and development of diabetes mellitus, and the associated cardiovascular complications, are known to exist. Premenopausal women are significantly less likely to develop these conditions relative to men, or women after menopause [3,4]. Accumulating data suggest that estrogens, particularly 17-beta estradiol which is the predominant and biologically active estrogen in premenopausal women [5], exert a protective effect in pancreatic beta cell health and function [6,7,8,9,10]. However, the mechanisms underlying this protection are still unknown.

Chronic hyperglycemia promotes insulin biosynthesis in pancreatic beta cells. Secreted proteins, like insulin, are co-translationally translocated and folded in the endoplasmic reticulum (ER). If increased protein demand overloads the ER, and the folding capacity of the ER is exceeded, unfolded or misfolded proteins can accumulate causing a condition known as ER stress [11]. Cells have developed mechanisms to restore ER homeostasis through the activation of the adaptive unfolded protein response (UPR) [12]. The adaptive UPR relieves ER stress by increasing protein folding capacity by inducing the synthesis of molecular chaperones and/or increasing the ER associated degradation (ERAD) of terminally misfolded proteins [12]. When there is protracted ER stress and homeostasis cannot be restored, the apoptotic UPR is activated, increasing the synthesis of pro-apoptotic pathways to eliminate the cell [12,13]. Secretory cells, such as pancreatic beta cells, traffic a relatively high percentage of protein synthesis to the ER and are therefore susceptible to ER stress. Several studies have shown an association between diabetes/hyperglycemia and pancreatic beta cell ER stress and apoptosis [11,14,15,16].

In this study, we investigate the effect of 17-beta estradiol on the beta cell UPR using a mouse model of hyperglycemia-induced atherosclerosis, the ApoE^−/−^:Ins2^+/Akita^ mouse, which exhibits sexual dimorphism in terms of glucose regulation and atherosclerosis progression [17]. Male ApoE^−/−^:Ins2^+/Akita^ mice are chronically hyperglycemic, whereas female ApoE^−/−^:Ins2^+/Akita^ mice are transiently hyperglycemic, with blood glucose levels normalizing by five weeks of age, which represents the time when they reach sexual maturity. Ovariectomized female ApoE^−/−^:Ins2^+/Akita^ mice are chronically hyperglycemic; however, supplementation with exogenous 17-beta estradiol restores glucose homeostasis [18]. To conduct mechanistic studies on the role of 17-beta estradiol in modulating the UPR response, we also use a murine pancreatic beta cell line, BTC6, able to secrete insulin in response to increasing glucose concentrations [19]. 

We hypothesize that 17-beta estradiol protects pancreatic beta cell health and function by modulating the UPR specifically by enhancing the adaptive UPR and by repressing the apoptotic UPR.

## 2. Results

### 2.1. UPR Activation in Isolated Pancreatic Islets from ApoE^−/−^ and ApoE^−/−^:Ins2^+/Akita^ Mice

**Figure 1:**
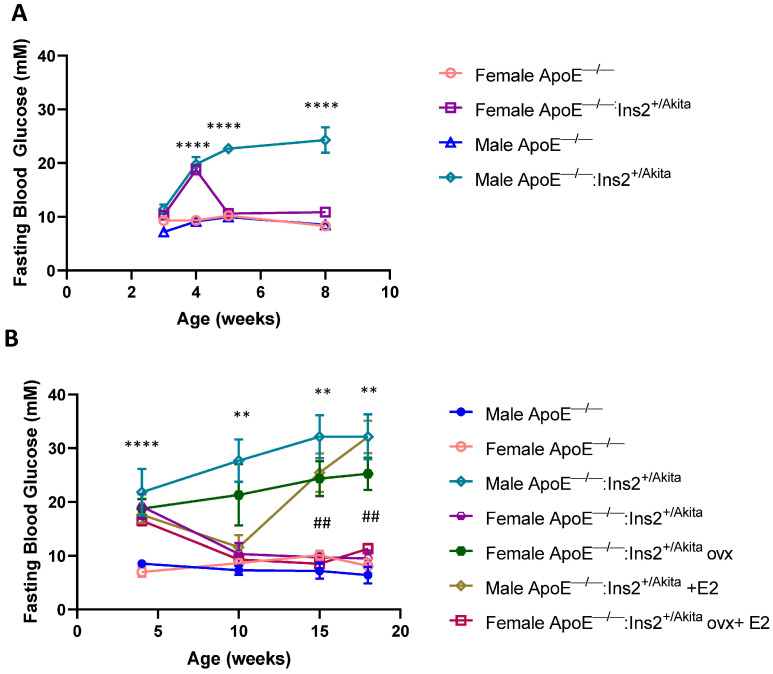
(**A**) Fasting blood glucose levels are lower in female ApoE^−/−^:Ins2^+/Akita^ mice, compared to age-matched male ApoE^−/−^:Ins2^+/Akita^ mice. Fasting blood glucose levels were determined in female and age-matched male ApoE^−/−^ and ApoE^−/−^:Ins2^+/Akita^. n = 4–7 mice per experimental group; female male ApoE^−/−^:Ins2^+/Akita^ mice. Bars represent standard error of the mean (SEM). (**B**) Ovariectomy increases and estrogen supplementation decreases fasting blood glucose levels in female ApoE^−/−^:Ins2^+/Akita^ mice. Fasting blood glucose levels were determined in sham operated female, ovariectomized (ovx) female, ovx female supplemented with 17-beta estradiol (E2), age-matched male ApoE^−/−^:Ins2^+/Akita^ mice supplemented with estrogen or not, and male and female ApoE^−/−^ controls. A transient reduction in blood glucose was observed in age-matched male ApoE^−/−^:Ins2^+/Akita^ mice supplemented with estrogen. n = 5 mice per experimental group. **** *p* < 0.0001 male ApoE^−/−^:Ins2^+/Akita^ mice vs. age matched male and female ApoE^−/−^ controls. ** *p* < 0.01 male ApoE^−/−^:Ins2^+/Akita^ mice vs. age matched sham operated female ApoE^−/−^:Ins2^+/Akita^ mice. ## *p* < 0.01 ovx female ApoE^−/−^:Ins2^+/Akita^ mice vs. ovx female ApoE^−/−^:Ins2^+/Akita^ mice + E2. Bars represent standard error of the mean (SEM).

We previously reported sex differences in the control of glucose homeostasis in the ApoE^−/−^:Ins2^+/Akita^ mouse model [18], with male mice developing chronic hyperglycemia, and female mice showing normalized glucose levels by five weeks of age, corresponding to the age when they reach sexual maturity (Figure 1).

**Figure 2:**
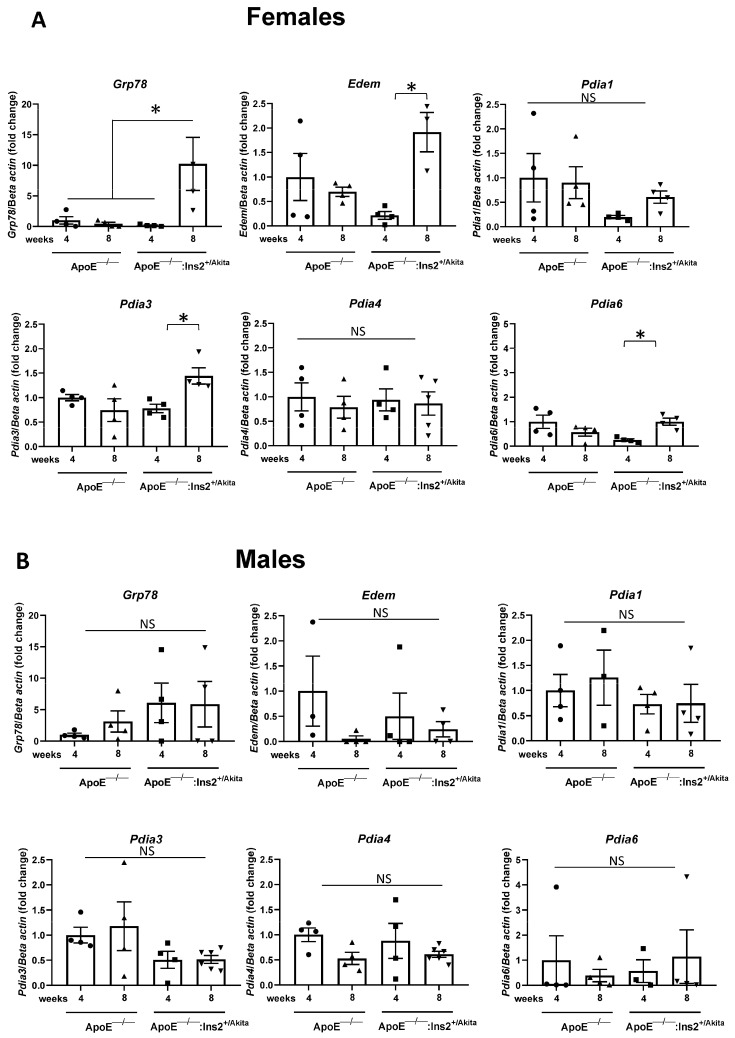

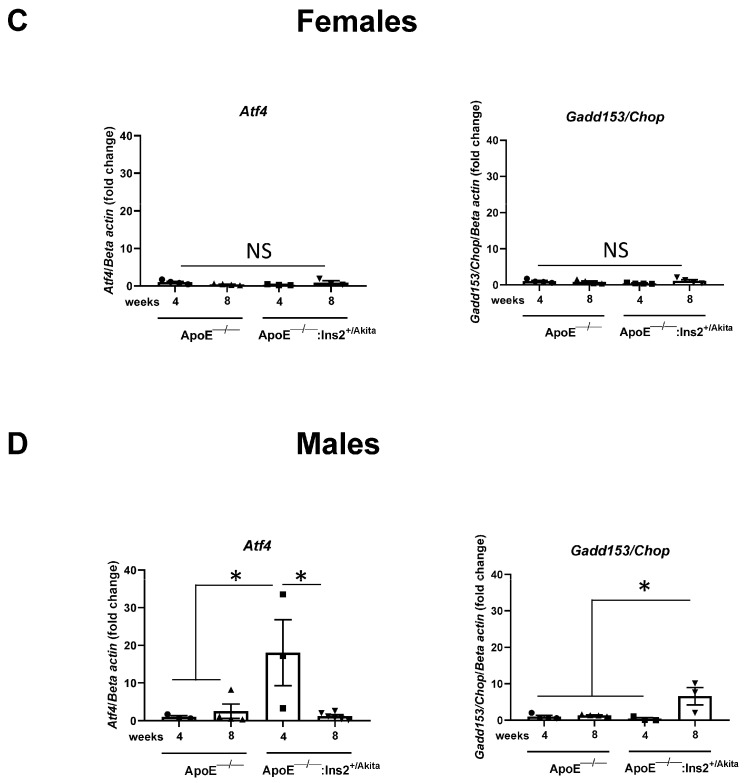
Expression of adaptive and apoptotic UPR markers in RNA transcripts of isolated pancreatic islets. Transcripts from isolated pancreatic islets were analyzed for the expression of the adaptive UPR markers *Grp78*, *Edem*, *Pdia1*, *Pdia3*, *Pdia4*, *Pdia6* in (**A**) female and (**B**) male ApoE^−/−^ and ApoE^−/−^:Ins2^+/Akita^ mice at 4 and 8 weeks of age. Expression of the apoptotic UPR markers *Gadd153/Chop* and *Atf4* in transcripts from isolated pancreatic islets of female (**C**) and male (**D**) ApoE^−/−^ and ApoE^−/−^:Ins2^+/Akita^ mice at 4 and 8 weeks of age. RNA transcripts isolated from pancreata from female ApoE^−/−^:Ins2^+/Akita^ mice show significantly increased expression of adaptive UPR markers, while transcripts from male ApoE^−/−^:Ins2^+/Akita^ mice show a significant increase in apoptotic UPR markers. Each sample represents pooled islets from n = 4–8 mice. n = 3–6 samples per experimental group, analyzed in technical duplicates. * *p* < 0.05, NS, not significant. Bars represent standard error of the mean (SEM).

To determine if these changes in glucose regulation correspond to modifications in the activation of the UPR, pancreatic islets were isolated from four and eight week old male and female ApoE^−/−^:Ins2^+/Akita^ mice and ApoE^−/−^ controls. Four weeks of age corresponds to a time where both male and female ApoE^−/−^:Ins2^+/Akita^ mice are hyperglycemic, and eight weeks of age corresponds to a time when male ApoE^−/−^:Ins2^+/Akita^ mice are hyperglycemic whereas glucose levels have normalized in females. Total RNA was isolated from the islets and the expression of specific UPR genes was quantified. We observed that 8 week old female ApoE^−/−^:Ins2^+/Akita^ mice present with a significant increase in the expression of markers of the adaptive UPR, compared to four week old female ApoE^−/−^:Ins2^+/Akita^ mice or age matched ApoE^−/−^ controls (Figure 2). The adaptive markers include *Grp78*, an ER resident chaperone involved in protein folding [20], and ER-associated protein degradation-enhancing alpha-mannosidase-like protein (*Edem*), which is involved in the process of endoplasmic reticulum-associated protein degradation (ERAD) [21]. In addition, protein disulfide isomerase (*Pdi*) isoforms *Pdia1*, *Pdia3*, *Pdia4*, *Pdia6*, which are known to play a role in cell survival under conditions of ER stress, were analyzed [22]. *Pdia3* and *Pdia6* are also significantly induced in 8 week old female ApoE^−/−^:Ins2^+/Akita^ mice relative to 4 week old female mice. No significant changes were detected in the expression of the examined adaptive UPR markers from male ApoE^−/−^:Ins2^+/Akita^ mice (Figure 2). In contrast, the markers of the apoptotic UPR, *Atf4* and *Gadd153/Chop*, are not induced in female ApoE^−/−^:Ins2^+/Akita^ mice (Figure 2), but are significantly increased in male ApoE^−/−^:Ins2^+/Akita^ mice at 4 and 8 weeks of age, respectively (Figure 2).

### 2.2. UPR Activation in Pancreatic Islets from ApoE^−/−^ and ApoE^−/−^:Ins2^+/Akita^ Mice

We previously reported the effects of estrogen depletion and 17-beta estradiol supplementation on glucose regulation in the ApoE^−/−^:Ins2^+/Akita^ mouse model [18]. Specifically, ovariectomized female ApoE^−/−^:Ins2^+/Akita^ mice remain chronically hyperglycemic, compared to sham operated female ApoE^−/−^:Ins2^+/Akita^ mice and ApoE^−/−^ controls further confirmed in Figure 1. Ovariectomized female ApoE^−/−^:Ins2^+/Akita^ mice supplemented with 17-beta estradiol have normalized blood glucose levels, compared to sham operated female ApoE^−/−^:Ins2^+/Akita^ mice and ApoE^−/−^ controls. Blood glucose levels are only transiently improved in male ApoE^−/−^:Ins2^+/Akita^ mice supplemented with 17-beta estradiol. In our previous study, we reported that the changes in fasting blood glucose levels are consistent with differences in pancreatic beta cell mass and glucose tolerance [18]. Specifically, ovariectomized female ApoE^−/−^:Ins2^+/Akita^ mice show a significant reduction in pancreatic beta cell mass and impaired glucose tolerance, compared to sham operated female ApoE^−/−^:Ins2^+/Akita^ mice, and 17-beta estradiol supplementation is able to rescue this phenotype. Male ApoE^−/−^:Ins2^+/Akita^ mice supplemented with 17-beta estradiol do not preserve beta cell mass; however, glucose tolerance is improved, compared to male ApoE^−/−^:Ins2^+/Akita^ mice not supplemented with 17-beta estradiol.

Metabolic parameters are consistent with our previous analysis, as we observed no significant differences in body weight across the male experimental groups as well as the female experimental group (Table 1 and Table 2) [18].

**Figure 3:**
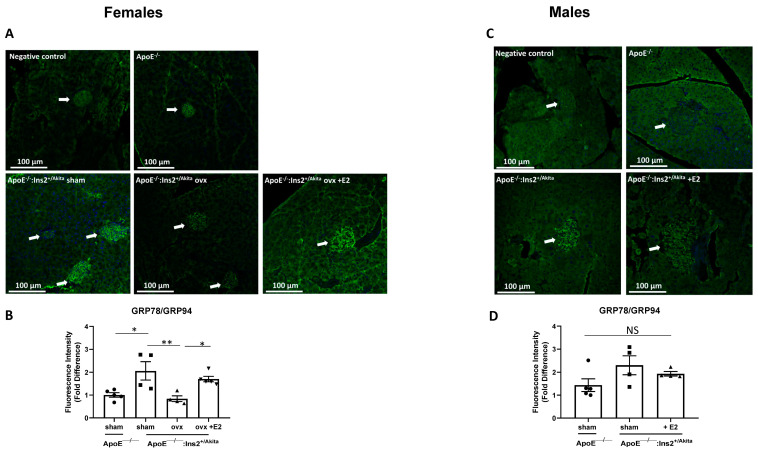
Expression of adaptive UPR markers GRP78/94 in pancreatic sections. Immunofluorescence staining was performed to measure the expression of adaptive UPR markers GRP78/GRP94 in pancreatic islet sections of sham-operated, ovariectomized, ovariectomized supplemented with 17-beta estradiol (E2) female (**A**,**B**) ApoE^−/−^:Ins2^+/Akita^ and female ApoE^−/−^ mice; and male (**C**,**D**) male ApoE^−/−^:Ins2^+/Akita^ supplemented with estrogen (E2) or not. Ovariectomy significantly reduces the expression of GRP78/94 in female ApoE^−/−^:Ins2^+/Akita^ mice, while no significant differences are observed in male experimental groups. n = 4–5 per group. * *p* < 0.05, ** *p* < 0.01, NS, not significant. Bars represent standard error of the mean (SEM).

**Figure 4:**
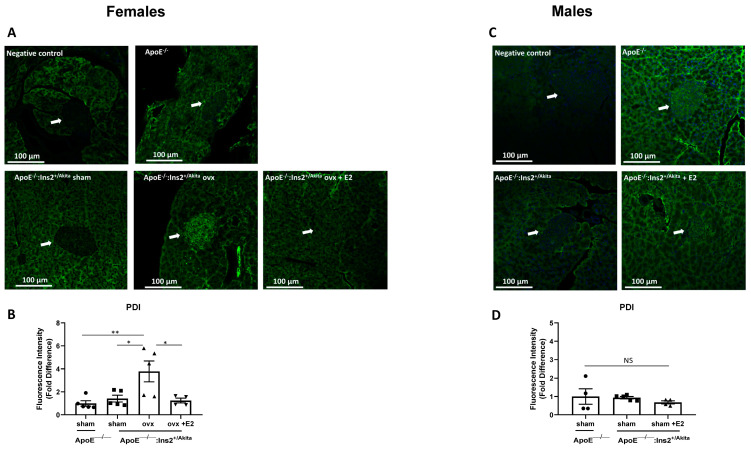
Expression of adaptive UPR marker PDI in pancreatic islets. Immunofluorescent staining was performed to evaluate the expression of adaptive UPR marker PDI in pancreatic islet sections of sham-operated, ovariectomized, ovariectomized supplemented with 17-beta estradiol (E2) female (**A**,**B**) ApoE^−/−^:Ins2^+/Akita^ and age matched female ApoE^−/−^ mice; and male (**C**,**D**) male ApoE^−/−^:Ins2^+/Akita^ supplemented with 17-beta estradiol or not. n = 4–5 per group. * *p* < 0.05, ** *p* < 0.01, NS, not significant. Bars represent standard error of the mean (SEM).

To determine the effects of 17-beta estradiol supplementation on UPR activation, male and ovariectomized female ApoE^−/−^:Ins2^+/Akita^ mice were implanted with a slow release 17-beta estradiol pellet at four weeks of age and pancreata were harvested at eighteen weeks of age. The expression of the adaptive UPR markers GRP78/GRP94 in sham operated female and ovariectomized female ApoE^−/−^:Ins2^+/Akita^ mice supplemented with 17-beta estradiol is significantly increased, compared to ovariectomized female ApoE^−/−^:Ins2^+/Akita^ mice (Figure 3). No significant differences are observed in male ApoE^−/−^:Ins2^+/Akita^ mice. PDI expression was significantly induced in the ovariectomized female ApoE^−/−^:Ins2^+/Akita^ mice, compared to the other female experimental groups (Figure 4), whereas no significant differences were observed in the expression of this adaptive UPR marker across the male experimental groups. 

**Figure 5:**
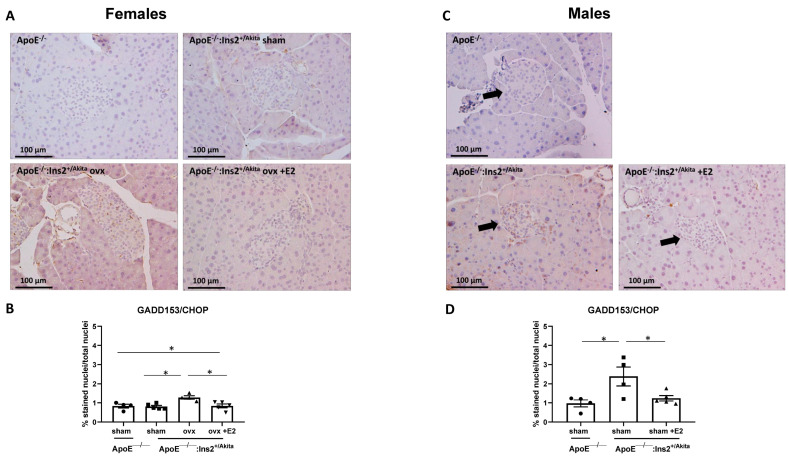
Expression of apoptotic UPR marker GADD153/CHOP in pancreatic sections. Immunostaining was performed to measure the expression of apoptotic UPR marker GADD153/CHOP in pancreatic islet sections of sham-operated, ovariectomized, ovariectomized supplemented with 17-beta estradiol (E2) female (**A**,**B**) ApoE^−/−^:Ins2^+/Akita^ and female ApoE^−/−^ mice; and male (**C**,**D**) ApoE^−/−^:Ins2^+/Akita^ supplemented with estrogen (E2) or not, compared to age matched ApoE^−/−^ controls. Data are expressed as percentage of brown-stained nuclei versus total nuclei in the islet. Ovariectomy significantly increases the expression of the apoptotic marker GADD153/CHOP in female ApoE^−/−^:Ins2^+/Akita^ mice to levels similar to those observed in male ApoE^−/−^:Ins2^+/Akita^ mice. n = 4–5 per group. * *p* < 0.05, Bars represent standard error of the mean (SEM).

The apoptotic UPR marker ATF4 was significantly increased in the ovariectomized female ApoE^−/−^:Ins2^+/Akita^ mice, but supplementation with17-beta estradiol in ovariectomized female ApoE^−/−^:Ins2^+/Akita^ mice significantly downregulates the expression of this marker to levels comparable to sham operated ApoE^−/−^:Ins2^+/Akita^ mice and the ApoE^−/−^ controls (Supplementary Figure S1). No significant differences in ATF4 expression were observed in male ApoE^−/−^:Ins2^+/Akita^ mice in the presence or absence of supplemented 17-beta estradiol, relative to the ApoE^−/−^ controls. Similarly to what was observed with ATF4, the expression of GADD153/CHOP is significantly induced in the ovariectomized female ApoE^−/−^:Ins2^+/Akita^ mice (Figure 5). Ovariectomized female ApoE^−/−^:Ins2^+/Akita^ mice supplemented with 17-beta estradiol do not show an increase in the expression of this apoptotic UPR marker. The expression of GADD153/CHOP was significantly higher in male ApoE^−/−^:Ins2^+/Akita^ mice compared to ApoE^−/−^ controls. Supplementation with 17-beta estradiol reduced GADD153/CHOP to levels comparable to ApoE^−/−^ controls.

### 2.3. Effects of 17-Beta Estradiol in the Modulation of the Adaptive UPR in a Pancreatic Beta Cell Line 

To determine if 17-beta estradiol has an effect on BTC6 viability, cells were exposed to increasing concentrations of this hormone. There were no significant differences in cell viability for any concentrations analyzed (Supplementary Figure S2), therefore a concentration of 1 µM 17-beta estradiol was selected for further analysis.

To explore the mechanistic aspect of the effects of 17-beta estradiol in the presence of ER stress, BTC6 cells were pretreated with 17-beta estradiol for 24 h and subsequently challenged for 4 or 8 h with ER stress inducers; glucose (increasing concentrations of 11 mM, 25 mM, and 35 mM), tunicamycin (0.125 µM), or thapsigargin (0.25 µg/mL). Mannitol (30 mM) was used as an osmotic control. Cells were exposed to ER stress inducers for 4 or 8 h to evaluate the effects of these treatments on both adaptive and apoptotic UPR activation. We previously observed that BTC6 cells exposed to high glucose concentrations showed increased expression of UPR markers (Supplementary Figure S3).

**Figure 6:**
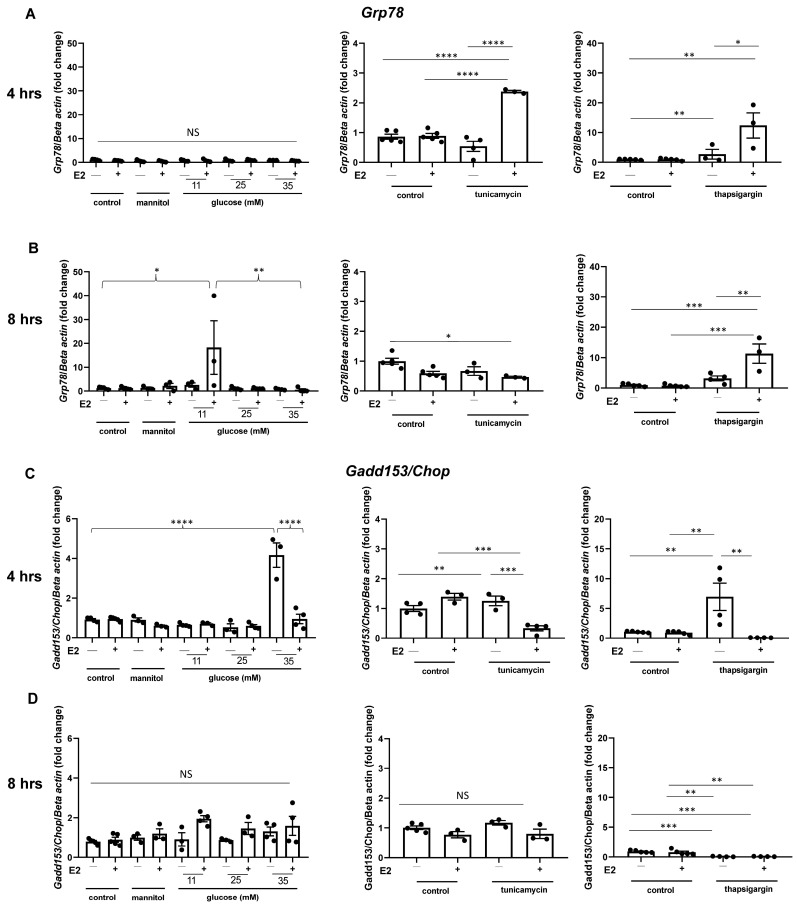
Expression of *Grp78* and *Gadd153/Chop* in BTC6 cells pretreated with estrogen. Transcripts from BTC6 cells pretreated with 17-beta estradiol (E2) were analyzed for expression of the adaptive UPR marker *Grp78* after (**A**) 4 h or (**B**) 8 h of exposure to glucose (11 mM, 25 mM, 35 mM), tunicamycin (0.125 µg/mL), or thapsigargin (0.25 µM). Mannitol (30 mM) was used as osmotic control. Transcripts of similarly treated BTC6 cells were analyzed for the expression of the apoptotic UPR marker *Gadd153/Chop* after (**C**) 4 h or (**D**) 8 h of exposure to ER stress. Exposure to 17-beta estradiol promotes an adaptive UPR response and reduces apoptotic UPR activation. n = 3–5 samples per experimental group, each sample has been analyzed in duplicate. * *p* < 0.05, ** *p* < 0.01, *** *p* < 0.001, **** *p* < 0.0001, NS, not significant. Bars represent standard error of the mean (SEM).

After 8 h of exposure to 11 mM glucose, 17-beta estradiol pretreatment significantly increases the expression of *Grp78* in BTC6 cells (Figure 6). In cells challenged with ER stress inducers tunicamycin and thapsigargin, 17-beta estradiol treatment increases the expression of this adaptive UPR marker after 4 h of exposure. At 8 h exposure, *Grp78* is still significantly induced in cells supplemented with 17-beta estradiol and exposed to thapsigargin; however, a reduction in this marker’s expression can be observed in cells pretreated with 17-beta estradiol and challenged with tunicamycin.

The expression of other adaptive UPR markers such as the *Pdi* isoforms (*Pdia1*, *Pdia3*, *Pdia4*, *Pdia6*) and *Edem* was also analyzed (Supplementary Figures S4–S8). *Pdia1* and *Pdia3* isoforms are significantly induced in cells pretreated with 17-beta estradiol and exposed to 25 mM glucose, tunicamycin and thapsigargin. Glucose did not induce the expression of the *Pdia4* isoform whereas a significant increase can be observed in cells pretreated with 17-beta estradiol and challenged with tunicamycin at 4 h of exposure, and with thapsigargin at both time points analyzed. 

17-beta estradiol significantly increases the expression of the *Pdia6* isoform in cells exposed to 11 mM glucose or after 4 h of exposure to thapsigargin, relative to those not receiving the hormone pretreatment. Tunicamycin does not seem to induce the expression of *Pdia6* isoforms.

No significant differences in the expression of *Edem* were observed in pancreatic BTC6 cells challenged with increasing glucose concentrations or tunicamycin. After 8 h of exposure, *Edem* was significantly induced in cells challenged with thapsigargin; however, 17-beta estradiol did not appear to have any effect on this induction.

### 2.4. Effects of 17-Beta Estradiol in the Modulation of the Apoptotic UPR in a Pancreatic Beta Cell Line 

To assess the effects of 17-beta estradiol on the apoptotic UPR, the expression of *Atf4* and *Gadd153/Chop* was analyzed. Increasing glucose concentrations, tunicamycin, and thapsigargin do not induce significant differences in the expression of the apoptotic UPR marker *Atf4* after 4 h of exposure (Supplementary Figure S9). After 8 h, 17-beta estradiol pretreatment significantly increases the expression of *Atf4* in pancreatic BTC6 cells challenged with 25 mM glucose. ATF4 is significantly increased in pancreatic beta TC6 cells challenged with thapsigargin for 8 h, compared to the controls and to cells pretreated with 17-beta estradiol and challenged with this ER stress inducer.

The expression of the apoptotic UPR marker *Gadd153/Chop* is significantly reduced by pretreatment with 17-beta estradiol in pancreatic BTC6 cells exposed to 35 mM glucose after 4 h of exposure (Figure 6). No significant differences in the expression of *Gadd153/Chop* are observed after 8 h of exposure to increasing glucose concentrations. 17-beta estradiol pretreatment significantly reduces the expression of *Gadd153/Chop* after 4 h of exposure to both tunicamycin and thapsigargin, relative to the controls.

## 3. Discussion

Sex differences in the development of diabetes do exist, with premenopausal women appearing to be protected from diabetes compared to men and postmenopausal women. This suggests that estrogens, and perhaps other sex hormones, play a protective role [23]. At least part of this protective effect appears to be linked to estrogen’s ability to modulate the UPR and alleviate ER stress, a condition that can be found in pancreatic beta cells under conditions of chronic hyperglycemia, such as diabetes [24]. We hypothesize that 17-beta estradiol, the predominant estrogen in premenopausal women, protects pancreatic beta cell health and function by enhancing the adaptive UPR and repressing the apoptotic UPR. 

To test this hypothesis we used a mouse model of hyperglycemia-induced atherosclerosis, the ApoE^−/−^:Ins2^+/Akita^ mouse. This mouse strain contains the Akita mutation, which is characterized by a point mutation in one allele of the Ins2 gene (C96Y) that leads to insulin misfolding [17]. This mouse model is notable because it shows sexual dimorphism in glucose regulation, with male ApoE^−/−^:Ins2^+/Akita^ mice remaining chronically hyperglycemic, and females being only transiently hyperglycemic [17,18]. Blood glucose levels in female ApoE^−/−^:Ins2^+/Akita^ mice normalize by five weeks of age which, as previously reported by our group, represents the time when these mice become sexually mature, and 17-beta estradiol and other estrogens’ levels increase (Figure 1) [18]. 

We quantified the expression of UPR genes in isolated pancreatic islets from male and female ApoE^−/−^:Ins2^+/Akita^ mice and age matched ApoE^−/−^ controls at four and eight weeks of age, as these ages bracket the observed normalization of blood glucose levels and the attainment of sexual maturity observed in female ApoE^−/−^:Ins2^+/Akita^ mice (Figure 2). We observed that the normalization of blood glucose levels in female ApoE^−/−^:Ins2^+/Akita^ mice is associated with the activation of the adaptive UPR. Specifically, there is an increase in the expression of ER resident chaperone *Grp78* involved in protein folding, *Edem* which is part of ERAD, and certain *Pdi* isoforms which are involved in the formation of disulfide bonds, a crucial step for the proper folding and processing of insulin. Conversely, in male ApoE^−/−^:Ins2^+/Akita^ mice, the adaptive UPR is not significantly induced, but there is a significant increase in the apoptotic UPR markers *Atf4* and *Gadd153/Chop*, compared to age-matched ApoE^−/−^ controls. These findings are consistent with previous observations showing that male ApoE^−/−^:Ins2^+/Akita^ mice have a significant loss of beta cell mass and are chronically hyperglycemic. This suggests that 17-beta estradiol levels could exert a protective effect on pancreatic beta cells preventing apoptosis by inhibiting the apoptotic UPR [18]. 

To investigate the effects of 17-beta estradiol depletion, mice were subjected to ovariectomy, a surgical procedure that significantly reduces the levels of circulating estrogens. Our results show that ovariectomy promotes a chronic hyperglycemic phenotype in female ApoE^−/−^:Ins2^+/Akita^ mice (Figure 1). Consistent with results on gene expression analysis in the isolated pancreatic islets, pancreatic sections show that GRP78/94 proteins are significantly induced in sham operated female ApoE^−/−^:Ins2^+/Akita^ mice, but not in the age-matched ovariectomized female ApoE^−/−^:Ins2^+/Akita^ mice (Figure 3). Pancreatic sections of male ApoE^−/−^:Ins2^+/Akita^ mice do not show significantly induced expression of GRP78/94, compared to age-matched controls. It is possible that the reduction in the expression of GRP78/94 in pancreatic sections of male and female ovariectomized ApoE^−/−^:Ins2^+/Akita^ mice may be a result of pancreatic islet damage as previously reported by Li et al., who observed that ApoE^−/−^ mice fed a high fat diet showed significant glucose impairment [25]. Further studies on this mouse model are needed to test this hypothesis. We also observed that the expression of PDI is significantly induced in the ovariectomized female ApoE^−/−^:Ins2^+/Akita^ mice, but not in the sham operated or ovariectomized female ApoE^−/−^:Ins2^+/Akita^ mice supplemented with 17-beta estradiol (Figure 4). This could be explained by the fact that PDI is directly involved in aiding in the formation of disulfide bonds, which is an important aspect of insulin processing and maturation. Furthermore, the antibody used is aimed at recognizing the PDI isoform PDIa1, which is known to play a role in proinsulin folding both in vivo and in vitro [22,26]. Ovariectomized female ApoE^−/−^:Ins2^+/Akita^ mice are hyperglycemic, therefore the demand for insulin is significantly increased, compared to that of normoglycemic sham operated female, or female ovariectomized ApoE^−/−^:Ins2^+/Akita^ mice supplemented with 17-beta estradiol. Hence, ovariectomized female ApoE^−/−^:Ins2^+/Akita^ mice likely require an increased expression of PDI to normalize blood glucose levels. These results, along with our in vitro observations on PDI, show that the expression of PDI is differentially regulated relative to other UPR markers. Further studies are needed to test this hypothesis. 

In terms of the apoptotic UPR, male ApoE^−/−^:Ins2^+/Akita^ mice do show a significant increase in the expression of GADD153/CHOP, compared to ApoE^−/−^:Ins2^+/Akita^ mice supplemented with 17-beta estradiol and ApoE^−/−^ controls (Figure 5). Similar to male ApoE^−/−^:Ins2^+/Akita^ mice, pancreatic sections of ovariectomized female ApoE^−/−^:Ins2^+/Akita^ mice show increased expression of the apoptotic UPR markers, ATF4 and GADD153/CHOP, but supplementation of 17-beta estradiol downregulates this expression. 

To more closely investigate the mechanisms and the pathways involved in this response, we carried out in vitro experiments in the cultured pancreatic beta cell line BTC6, exposing BTC6 cells to ER stress inducers for 4 or 8 h. Consistent with the in vivo findings, our studies in BTC6 cells show that 17-beta estradiol pretreatment significantly increases the expression of *Grp78* in cells challenged with 11 mM glucose for 8 h (Figure 6). Consistent with a previous study characterizing this cell line, we observed the maximum glucose stimulated insulin release from these cells in the presence of 11 mM glucose (Supplementary Figure S10) [27]. This suggests that the necessity of synthesizing increased quantities of insulin in response to higher glucose concentrations might induce a status of protracted ER stress, which can be rescued by 17-beta estradiol. In accordance with these findings, pre-treatment with 17-beta estradiol significantly reduces the expression of the apoptotic UPR marker *Gadd153/Chop* in cells challenged with all ER stress inducers (Figure 6). Taken together, these findings indicate that 17-beta estradiol enhances the adaptive UPR in short-term exposure and inhibits the activation of the apoptotic UPR in conditions of protracted ER stress after 8 h of exposure. 

Similar to what was observed in pancreatic islets, 17-beta estradiol significantly increased the expression of the isoforms *Pdia3* and *Pdia6*, which are known to play a role in contributing to cell survival under conditions of ER stress [22,28]. We also observed that *Pdia1*, which in vitro has shown to aid in proper maturation of proinsulin [26], was also increased in cells pretreated with 17-beta estradiol and challenged with 25 mM glucose (Supplementary Figure S4). In contrast to what was observed in isolated pancreatic islets, no significant differences were observed in *Edem* expression in BTC6 cells (Supplementary Figure S8). This could be explained by the fact that the nature of the ER stress differed in the islets, derived from the ApoE^−/−^:Ins2^+/Akita^ mice, compared to the BTC6 cell line, which does not present with the Akita mutation. Therefore, the amount of terminally misfolded insulin in the cell line is likely much less than what observed in mice, and not enough to trigger a significant increase in the ERAD. One limitation of this study was our inability to accurately measure insulin and C peptide levels in the ApoE^−/−^:Ins2^+/Akita^ mouse model. Several attempts, using different ELISA and HTRF systems were not successful. It is likely that the mutation typical of this strain interferes with the detection of insulin and C peptide.

To further investigate the role of estrogen in alleviating protein misfolding and confirm the role of *Pdi* observed in our animal and cell experiments, further analysis using computer assisted molecular modeling methods, biophysical techniques such as thioflavin (ThT) fluorescence, or nuclear magnetic resonance spectroscopy (NMR), could shed more light on the process of protein misfolding in diabetes and how estradiol can modulate the UPR to alleviate this condition [29,30,31,32]. These techniques could provide more information on the effects of estrogen by measuring endoplasmic reticulum stress in real time in the case of ThT fluorescence and characterize possible interactions and potential alterations in protein structure as is the case of NMR [29,30,31,32].

It is notable that relatively high levels of glucose (35 mM) are required to induce ER stress in the BTC6 cell line. We also used supraphysiological concentrations of 17-beta estradiol to ensure the observation of the effects of this hormone on UPR activation in response to ER stress. This is likely due, at least in part, to the acute nature of experiments in cultured cells as well as the features of this cell line [19,27]. One interesting observation from the in vitro experiments is that the pretreatment with 17-beta estradiol did not significantly affect UPR gene expression in the absence of ER stress. Thus, it appears that 17-beta estradiol may modulate an aspect of the UPR pathway. Future experiments will investigate the effects of 17-beta estradiol on the three main UPR regulators (inositol-requiring protein 1, IRE1, PKR-like endoplasmic reticulum kinase, PERK, and activating transcription factor-6, ATF-6), as well as an analysis of which specific estrogen receptors are involved in this process. These studies may also shed light on the reasons for the transient protection that 17-beta estradiol confers to male ApoE^−/−^:Ins2^+/Akita^ mice. 

It is important to note that obesity is a strong risk factor for that type 2 diabetes mellitus and studies have shown that obesity is associated with conditions of ER stress [33,34,35,36]. In a clinical study, adipose biopsies showed a significant increase in the expression of ER stress markers, and that these correlate with increased body mass indexes and percent fat [33]. Interestingly, a recent clinical study showed that bariatric surgery ameliorated the expression of ER stress markers [36]. Our findings may suggest a potential role for 17-beta estradiol in reducing ER stress by enhancing the adaptive UPR and restoring homeostasis in the pancreas as well as other tissues involved in glucose homeostasis such as the adipose tissue. Further studies will be required to confirm the modulation of ER stress in the adipose tissue by 17-beta estradiol.

It should also be noted that prolactin is involved in the upregulation of pancreatic beta cell mass, stimulating pancreatic beta cell replication in pancreatic islets as well as insulinoma cells [37,38,39]. It has been reported that ovariectomy can impair the secretion of prolactin [40]. Further studies should be conducted to evaluate whether the female ovariectomized ApoE^−/−^:Ins2^+/Akita^ mice show significant alterations in prolactin secretion, and whether this is associated with the loss of pancreatic beta cell mass and an impairment of glucose tolerance. In our previous analysis we observed that 17-beta estrogen supplementation in ovariectomized female ApoE^−/−^:Ins2^+/Akita^ mice maintains beta cell mass and improves glucose tolerance, compared to ovariectomized female ApoE^−/−^:Ins2^+/Akita^ mice [18]. 

Overall, our findings suggest that, under conditions that stimulate ER stress, such as chronic hyperglycemia, 17-beta estradiol exerts a protective effect by enhancing the adaptive UPR and repressing the apoptotic UPR. The results from our animal studies are consistent with various in vitro studies showing that 17-beta estradiol has a similar modulation of the adaptive and apoptotic UPR in different cell types. A study in a human gastric adenocarcinoma cell line treated with an ER stress inducer, tunicamycin, showed that those treated with 17-beta estradiol significantly reduced ER stress-induced apoptosis [41]. A similar result was also observed in another study using a mouse-derived osteoblast cell line, where ER stress was induced using another ER stress inducer, thapsigargin [42]. In this case, 17-beta estradiol was able to reduce ER stress by increasing the expression of the protein chaperone GRP78, as well as repressing apoptosis by inhibiting the caspase cascade. Finally, a study examining the pancreatic cell line INS1 showed that 17-beta estradiol was able to reduce the levels of ER stress in the presence of high glucose levels, protecting these cells from cell death [24]. 

## 4. Materials and Methods

Animal models

Male ApoE^−/−^:Ins2^+/Akita^ mice were crossed with female ApoE^−/−^:Ins2^+/+^ mice to create the experimental ApoE^−/−^:Ins2^+/Akita^ mouse strain. Genotypes were confirmed using PCR by methods previously described [17,18]. All experimental mice received a standard diet (2018 Teklad Global 18% Protein Rodent Diet, Harlan Teklad, Madison, WI, USA) ad libitum, with free access to water. Subsets of female ApoE^−/−^:Ins2^+/Akita^ mice underwent ovariectomy at 4 weeks of age with the experimental endpoint at 25 weeks of age. Additional subsets of female ApoE^−/−^:Ins2^+/Akita^ mice were ovariectomized at four weeks of age, and received a subcutaneous 17-beta estradiol pellet implant (0.1 mg, 90 days release, Innovative Research of America, Sarasota, FL, USA). A subset of male ApoE^−/−^:Ins2^+/Akita^ mice also received the 17-beta estradiol pellet implant at four weeks of age. The pellet was implanted subcutaneously on the lateral side of neck between the ear and the shoulder of the mouse. This pellet continuously releases 17-beta estradiol at a dose of 0.1 mg/pellet for 90 days allowing the hormone to circulate at a physiological range [43,44,45]. The experimental endpoint for mice receiving the 17-beta estradiol pellet and their respective controls was 18 weeks of age. All animal procedures were pre-approved by the McMaster University Animal Research Ethics Board.

Ovariectomy

Ovariectomies were performed using methods previously described [17,46]. Mice at four weeks of age (n = 5–10 per experimental group) were anesthetized using isoflurane (5% induction, 2.5% maintenance of anaesthesia). A 3 × 3 cm incision area surrounding the iliac crest was shaved and cleaned, and a midline horizontal incision through the skin was performed. The ovary was identified, and an incision was made through the muscle layer to reach the abdominal cavity. The ovary was pulled out by gently removing the surrounding fat pad from the abdominal cavity. The uterine horn and vessels were double ligated (0.7 cm and 1 cm distally from the ovary), and the ovary was excised. The remaining tissue was put back in the abdominal cavity, and the incision was sutured. The contralateral ovary was removed in a similar fashion. The skin wound was closed using a wound clipper. Sham operated animals (n = 5–10 per experimental group) received the same incisions and isolation of ovaries; however, ovaries were not removed.

Tissue harvesting

Mice were anesthetized with isoflurane and euthanized by cervical dislocation. Vasculature was rinsed with phosphate-buffered saline. Pancreata were collected and fixed in 10% neutral-buffered formalin and stored at room temperature.

Analysis of the pancreas

Pancreata from male and female ApoE^−/−^ and ApoE^−/−^:Ins2^+/Akita^ mice were harvested at 18 or 25 weeks of age. Immunohistochemical or immunofluorescent staining for the following UPR markers was performed using the indicated antibodies at the reported dilutions: glucose-regulated protein 78/94, GRP78/GRP94 (monoclonal mouse KDEL antibody, ADI-SPA-827-J, Enzo/Cedarlane, Burlington, ON, Canada, 1:250 dilution), growth arrest and DNA damage-inducible gene/C/EBP Homologous Protein, GADD153/CHOP (monoclonal mouse GADD153 (B3) sc-7531, Santa Cruz Biotechnology, Dallas, TX, USA, 1:50 dilution), protein disulfide isomerase, PDI (monoclonal mouse antibody, ADI-SPA-891-F, Enzo/Cedarlane, Burlington, ON, Canada, 1:200 dilution), activating transcription factor 4, ATF4 (polyclonal rabbit antibody, 10835-1-AP, Thermo Fischer, Mississauga, ON, Canada, 1:200 dilution). Secondary antibodies used were goat anti-mouse biotinylated IgM (BA 2020, Vector, Burlington, ON, Canada), Alexa Fluor 488 goat anti-mouse IgG (A11001, Thermo Scientific, Middletown, VA, USA), Alexa Fluor 488 goat anti-rabbit IgG (A11008, Thermo Scientific, Middletown, VA, USA), at a dilution of 1:200 each. All immunofluorescent staining experiments were counterstained with 4′-6′-diamidino-2-phenylindole, DAPI (Invitrogen, Carlsbad, CA, USA, 1:5000 dilution). Separate sections were stained with pre-immune IgG instead of the primary antibody, to control for non-specific staining. Immunohistochemistry used the developer reagent DAB+ Substrate Chromogen System (DAKO, K3468, Agilent Technologies, Santa Clara, CA, USA) as directed by the manufacturer.

Staining was performed on paraffin-embedded sections of pancreas (6 µm thick) from male and female ApoE^−/−^ and ApoE^−/−^:Ins2^+/Akita^ mice. The entire pancreas was analyzed at 180 µm intervals for a comprehensive overview of the islets within the organ. A total of six sections per mouse were used to perform immunohistochemical and immunofluorescent analysis, and a total of n = 12–20 islets per mouse were selected for the intensity of fluorescence analysis. A total of n = 5–8 mice per experimental group were analyzed. Immunofluorescence intensity was visualized, and images were captured using a Leica STELLARIS 5 confocal microscope. Analysis was performed using ImageJ software 1.53k (NIH, Bethesda, MD, USA; http://imagej.nih.gov/ij, accessed date 28 June 2021). The intensity of fluorescence staining for each mouse was calculated as follows: 

Intensity of fluorescence = (average intensity of fluorescence of the pancreatic islets per mouse adjusted for islet area) − (average intensity of fluorescence of the pancreatic islets of the negative control adjusted for islet area). 

Immunohistochemical analysis was captured using a Leitz LABORLUX S microscope connected to a DP71 Olympus camera. Quantification of GADD153/CHOP was performed by counting the brown-stained nuclei versus the total number of nuclei in the islet (n = 12–20 islets per mouse; n = 4–8 mice per experimental group).

Islet isolation

Pancreatic islet isolation was carried out using established methods [47,48]. Pancreata were excised from 4 or 8 week old mice and placed in collagenase (Collagenase type XI, C7657, Sigma Aldrich, Oakville, ON, Canada) at 37 °C for digestion, and hand shaken regularly for 20 min. Islets were isolated on a histopaque gradient (Histopaque 1119, 11,191 and Histopaque 1077, 10,771, Sigma Aldrich, Oakville, ON, Canada), and washed with Hank’s Balanced Salt Solution (HBSS). Each islet sample represents pooled islets from 4–8 mice pancreata. A total of n = 3–6 samples per experimental group were analyzed.

Cell line and cell experiments

Cell experiments were performed using the adherent pancreatic beta cell line TC6 (BTC6, CRL-11506, ATCC, Manassas, VA, USA), which is capable of secreting insulin in response to increasing glucose concentrations (Supplementary Figure S10) [19,27]. BTC6 cells were determined to be of female origin (Supplementary Figure S11). BTC6 cells were cultured in an incubator at 37 °C, 95% air, 5% CO_2_ using the ATCC-formulated Dulbecco’s Modified Eagle’s medium, DMEM (ATCC 30-2002, ATCC, Manassas, VA, USA) supplemented with 15% heat-inactivated Fetal Bovine Serum, FBS (F2242, Sigma Aldrich, Oakville, ON, Canada), and 1% penicillin-streptomycin (PenStrep, 10,000 U/mL, GIBCO, Thermo Scientific, Middletown, VA, USA). Cells were passaged using trypsin-EDTA 0.05% with phenol red (25300054, Thermo Scientific, Middletown, VA, USA). All experiments were conducted using cells between passages 4 and 10. To eliminate extraneous estrogen and estrogen-like effects, all experiments were performed using phenol red free DMEM (DMEM 4.5 mM glucose with L-glutamine, 30-2002, Cedarlane, Burlington, ON, Canada) supplemented with 15% charcoal-stripped (CS) FBS, (F6765-500ML, Sigma Aldrich, Oakville, ON, Canada), and 1% penicillin-streptomycin.

Cell viability was determined by plating BTC6 cells (cell seeding 100,000 cells per well) in a 96 well. Cells were cultured with phenol red free-CS-DMEM for three days and medium was replaced with fresh phenol red free-CS-DMEM containing increasing concentrations of 17-beta estradiol (E2758-250MG, Sigma Aldrich, Oakville, ON, Canada), and 10% *v*/*v* Alamar blue reagent (alamarBlue^®^, BUF012A, Bio-Rad, Mississauga, ON, Canada). Cells were placed in an incubator at 37 °C in humidified 95% air, 5% CO_2_. Cell viability was measured at 24 h using a spectrophotometer (SpectraMax Plus 384 Microplate Reader, Molecular Devices, San Jose, CA, USA) with excitation at 570 nm and emission at 600 nm. 

To determine the effects of 17-beta estradiol on UPR modulation, BTC6 cells were cultured in 24 well plates (cell seeding 300,000 cells/well) for three days with phenol red free-CS-DMEM in an incubator at 37 °C, 95% air, 5% CO_2_. Medium was replaced on the third day and cells were pretreated with 1 µM 17-beta estradiol in phenol red free-CS-DMEM for 24 h. Subsequently, subsets of cells were exposed to increased glucose concentrations (11 mM, 25 mM, or 35 mM), or ER stress inducers tunicamycin (0.125 µg/mL) or thapsigargin (0.25 µM), or the osmotic control mannitol (30 mM). Samples were collected after 4 and 8 h of exposure for gene expression analysis. For all in vitro experiments, n = 3–4 samples per experimental condition were used, and each sample was analyzed in duplicate.

A stock of 17-beta estradiol (100 mM) was diluted in 100% ethanol according to the manufacturer’s recommendations. Serial dilutions were prepared by diluting the 100 mM stock to 10 mM in 50% ethanol, and furthermore to 1 mM in sterile dH_2_O. Working concentrations of 1 µM 17-beta estradiol were prepared by serial diluting the 1 mM stock in phenol red-free-CS-DMEM. This method allows for ethanol concentration to be significantly reduced so that a vehicle control is not needed.

Gene expression analysis by qRT-PCR

Total RNA from pancreatic islets or harvested samples of BTC6 cells were isolated using TRIzol reagent (TRIzol^®^, 15596-018, Life Technologies, Burlington, ON, Canada). mRNA was reverse transcribed using the High-Capacity cDNA Reverse Transcription Kit (4368813, Applied Biosystems, Foster City, CA, USA). Real time PCR amplification was performed using SYBR green (SensiFAST™ SYBR Hi-ROX kit, Bioline, Wolston, Warrington, UK) reagent. Transcript amplification was normalized to the reference gene beta actin. Primers sequences are listed in Table 3. Analysis of housekeeping genes was performed using previously described methods [49]. *Beta actin* was selected as the reference gene as it presented the least variability among experimental groups, compared to other reference genes tested, *Cyclophilin A* and *Hprt1* (Supplementary Figures S12 and S13). Data were normalized to the internal control *Beta actin* and then further normalized to ApoE^−/−^ controls (for transcripts of isolated pancreatic islets), or the untreated control (for BTC6). Controls are set to “1.0”.

Each islet sample represents pooled islets from 4–8 mice pancreata. A total of n = 3–6 samples per experimental group were analyzed. For all qRT-PCR analysis, all biological replicates were analyzed in technical duplicates, and each biological replicate represents the average of the technical duplicates.

Statistical Analysis

Statistical analysis of multiple groups was assessed using t-test, one-way or two-way analysis of variance (ANOVA), where appropriate. Data are presented as mean ± standard error of the mean (SEM). Analyses were performed using ImageJ. A value of *p* < 0.05 was considered statistically significant.

## 5. Conclusions

Taken together, these results could provide a possible explanation of why pre-menopausal women have a lower risk of developing diabetes than men or post-menopausal women [3,24,41,42,50]. Further research will be needed to determine the specific molecular mechanisms by which 17-beta estradiol enhances the adaptive UPR and represses the apoptotic UPR under conditions of ER stress such as those observed during protracted hyperglycemia.

## Figures and Tables

**Table 1:** Male mouse body weights ± standard deviation, SD. n = 5 mice per experimental group.

ApoE^−/−^	ApoE^−/−^:Ins2^+/Akita^	ApoE^−/−^:Ins2^+/Akita^ + 17-Beta Estradiol
28.04 ± 0.63	25.70 ± 2.11	27.50 ± 1.77

**Table 2:** Female mouse body weights ± standard deviation, SD. n = 5 mice per experimental group.

ApoE^−/−^	ApoE^−/−^:Ins2^+/Akita^	ApoE^−/−^:Ins2^+/Akita^ Ovx	ApoE^−/−^:Ins2^+/Akita^ Ovx + 17-Beta Estradiol
20.94 ± 0.92	23.10 ± 2.38	23.18 ± 1.77	23.16 ± 2.58

**Table 3:** Mouse primer sequences for qRT-PCR.

Target	Forward (5′→3′)	Reverse (5′→3′)
Mouse *Beta actin*	GGCACCACACCTTTACAATG	GGGGTGTTGAAGGTCTCAAAC
Mouse *Cyclophilin A*	TGTGCCAGGGTGGTGACTTTAC	TGGGAACCGTTTGTGTTTGG
Mouse *Hprt1*	AGATGTCATGAAGGAGATGG	TACAGTAGCTCTTCAGTCTG
Mouse *Grp78*	CTGGGTACATTTGATCTGACTGG	GCATTCTGGTGGCTTTCCAGCCATTC
Mouse *Edem*	CTACCTGCGAAGAGGCCG	GTTCATGAGCTGCCCACTGA
Mouse *Pdia1*	CAAGATCAAGCCCCACCTGAT	AGTTCCCCCCAACCAGTACTT
Mouse *Pdia3*	GATGGAATTGTCAGCCACTTG	GGTGTGTGCAAATCGGTAGTT
Mouse *Pdia4*	AGCTCCTTGGCAGCTTTCTC	TGCAGACATTATTTTGGTGGA
Mouse *Pdia6*	CTAGCAGTCAGCGGTCTGTAT	CACAGGCCGTCACTCTGAAT
Mouse *Atf4*	ATGGCCGGCTATGGATGAT	CGAAGTCAAACTCTTTCAGATCCATT
Mouse *Gadd153/Chop*	TATCTCATCCCCAGGAAACG	CTGCTCCTTCTCCTTCATGC

## Data Availability

Supplementary data can be found at DOI 10.6084/m9.figshare.24201456 (https://figshare.com/articles/figure/supplementary_figures_De_Paoli_2023_pdf/24201456, accessed on 19 January 2024).

## Supplementary Materials

The following supporting information can be downloaded at: https://www.mdpi.com/article/10.3390/ijms25031816/s1.
