# Supplementary material for: Investigating the Role of 17-Beta Estradiol in the Regulation of the Unfolded Protein Response (UPR) in Pancreatic Beta Cells
Source: Int J Mol Sci. 2024 Feb 2;25(3):1816. doi: 10.3390/ijms25031816 (PMC10855194; doi:10.3390/ijms25031816)
Supplement: Supplementary file 1 [file ijms-25-01816-s001.zip › ijms-2744717-supplementary.pdf]

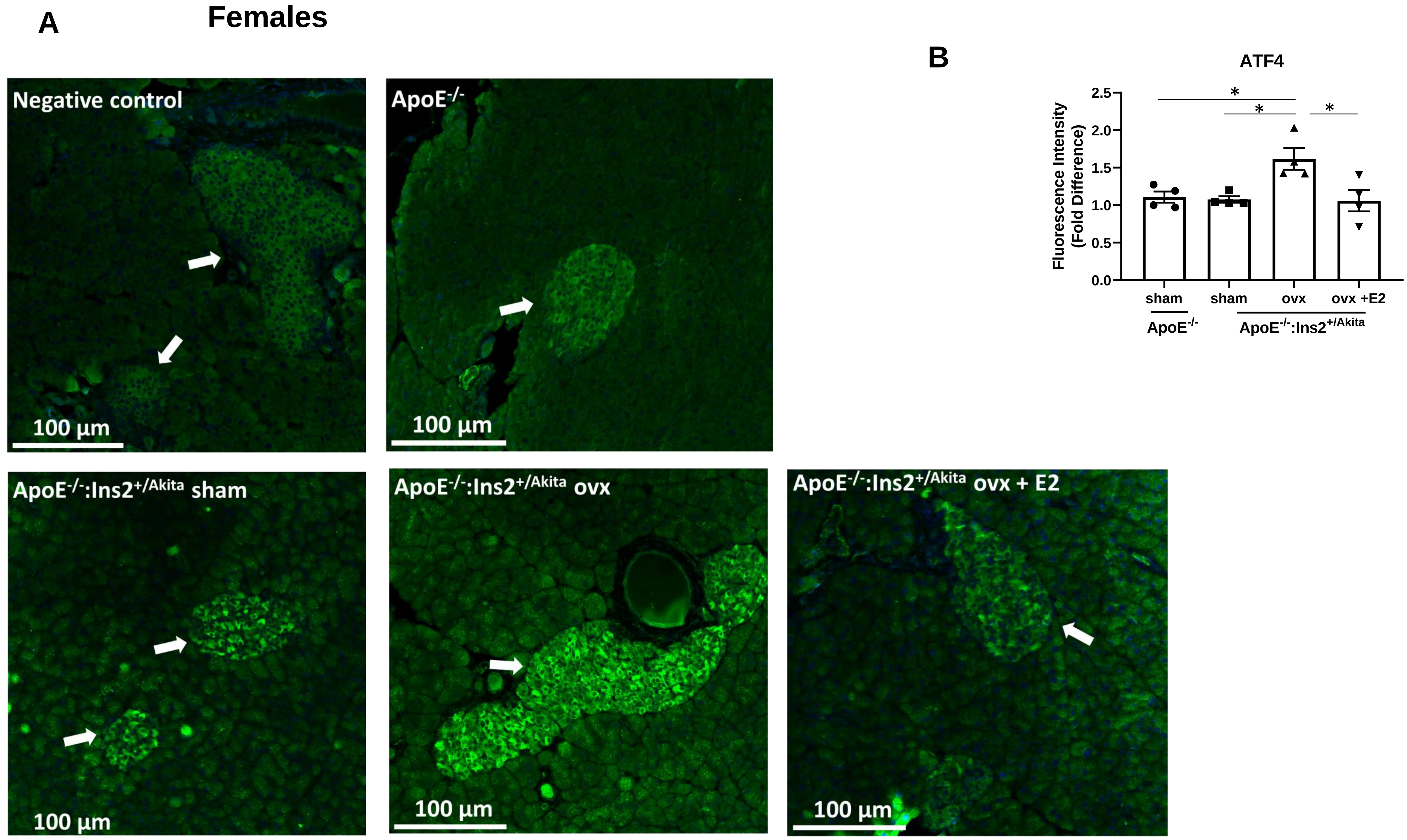
A
Females
Negative control
100 µm
ApoE-/-
100 µm
ApoE-/-:Ins2+/Akita sham
100 µm
ApoE-/-:Ins2+/Akita ovx
100 µm
ApoE-/-:Ins2+/Akita ovx + E2
100 µm
B
ATF4
Fluorescence Intensity (Fold Difference)
2.5
2.0
1.5
1.0
0.5
0.0
*
*
*
sham
sham
ovx
ovx +E2
ApoE-/-
ApoE-/-:Ins2+/Akita

Males

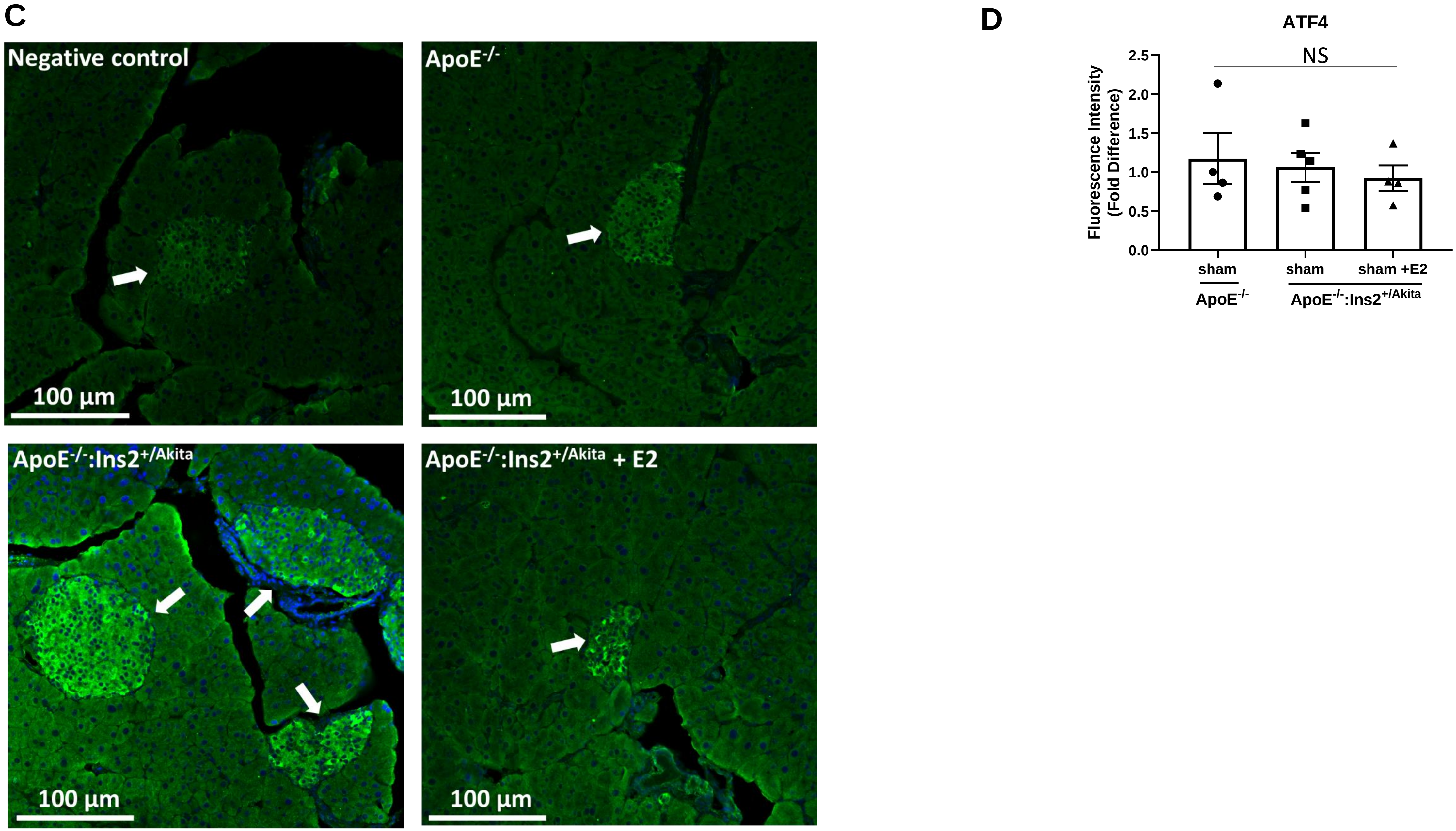
C
Negative control
ApoE-/-
ApoE-/-:Ins2+/Akita
ApoE-/-:Ins2+/Akita + E2
100 µm
100 µm
100 µm
100 µm
D
ATF4
NS
Fluorescence Intensity (Fold Difference)
2.5
2.0
1.5
1.0
0.5
0.0
sham
sham
sham +E2
ApoE-/-
ApoE-/-:Ins2+/Akita

Supplementary Figure S1. Expression of apoptotic UPR marker ATF4 in pancreatic islets. Immunofluorescent staining was performed to measure the expression of apoptotic UPR marker ATF4 in pancreatic islet sections of sham-operated, ovariectomized, ovariectomized supplemented with 17-beta estradiol (E2) female **(A, B)** ApoE$^{-/-}$:Ins2$^{+/Akita}$ and female ApoE$^{-/-}$ mice; and male **(C, D)** ApoE$^{-/-}$:Ins2$^{+/Akita}$ supplemented with 17-beta estradiol or not, compared to age matched ApoE$^{-/-}$ controls. n=4-5 per group. *p<0.05, NS, not significant. Bars represent standard error of the mean (SEM).

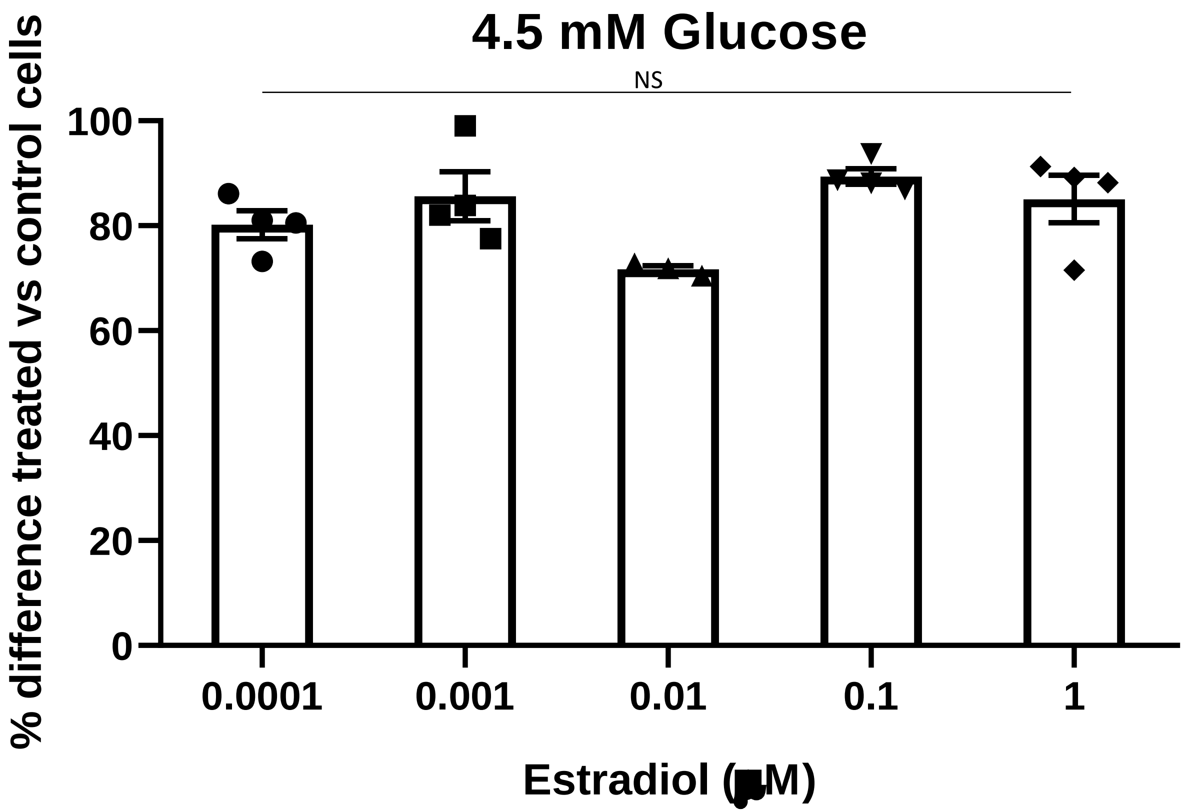

Supplementary Figure S2. BTC6 cell viability in cells treated with 17-beta estradiol. BTC6 cells were treated with increasing concentrations of 17-beta estradiol for 24 hours in charcoal-stripped, phenol red-free DMEM containing 10%v/v alamarBlue reagent. Cell viability was calculated with a spectrophotometer after the 24 hour exposure. n=4 per group, analyzed in duplicates. NS, not significant. Bars represent standard error of the mean (SEM).

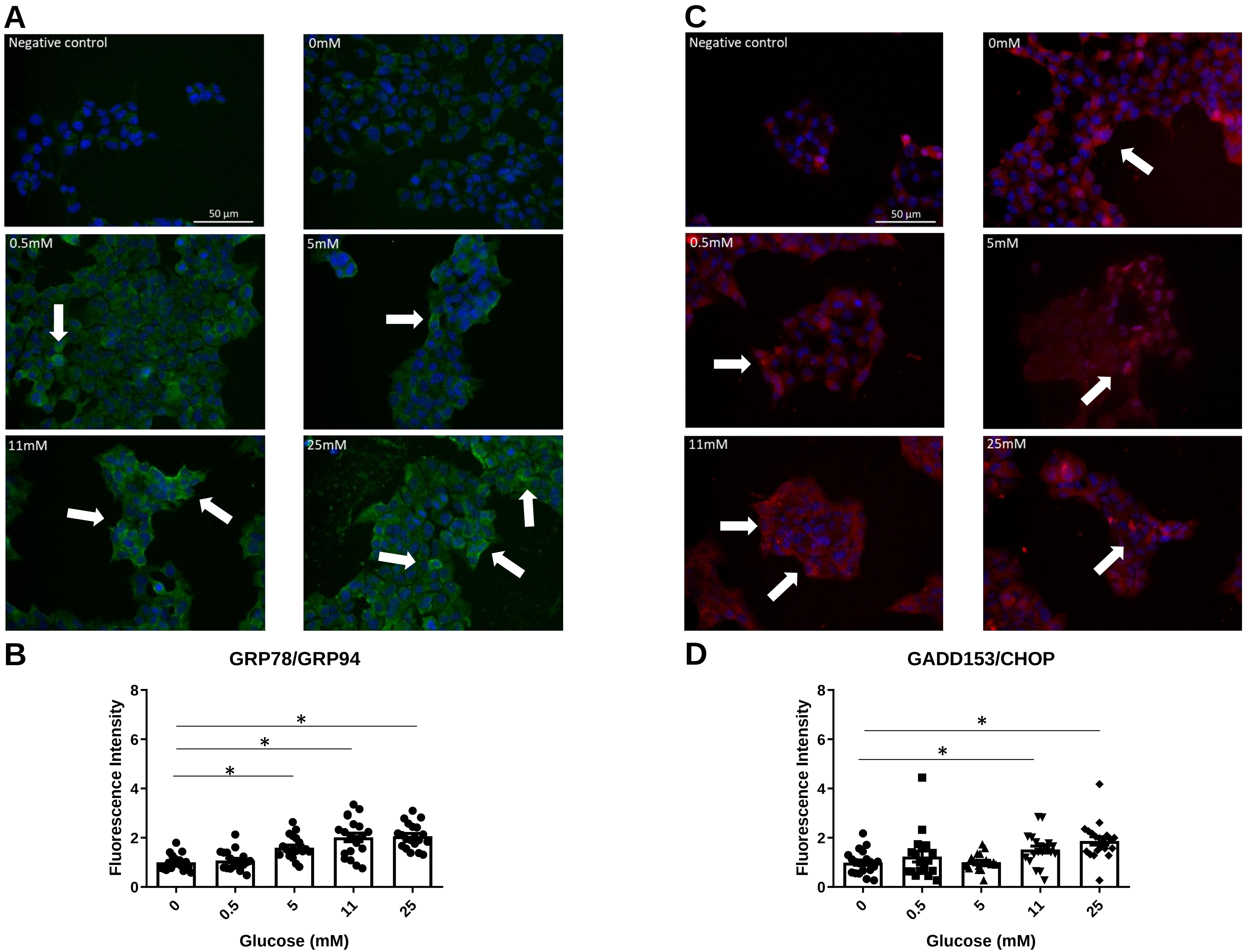
A
Negative control
50 µm
0mM
0.5mM
5mM
11mM
25mM
C
Negative control
50 µm
0mM
0.5mM
5mM
11mM
25mM
B
GRP78/GRP94
Fluorescence Intensity
0
2
4
6
8
*
*
*
0
0.5
5
11
25
Glucose (mM)
D
GADD153/CHOP
Fluorescence Intensity
0
2
4
6
8
*
*
0
0.5
5
11
25
Glucose (mM)

Supplementary Figure S3. UPR activation in BTC6 cells exposed to increasing glucose concentrations. BTC6 cells were exposed for two hours to Krebs-Ringer HEPES buffer containing increasing concentrations of glucose (0-25 mM). Immunofluorescence was determined by assessing fluorescence intensity of cells using an antibody against GRP78/GRP94 (monoclonal mouse KDEL antibody, ADI-SPA-827-J, Enzo/Cedarlane, Burlington, ON, Canada),1:150 dilution, or an antibody against GADD153/CHOP (monoclonal mouse GADD153 (B3) sc-7531, Santa Cruz Biotechnology, Dallas, TX, USA), 1:50 dilution, and and counterstained with DAPI (Invitrogen, Carlsbad, CA) at a dilution of 1:5,000. Immunostaining was detected using secondary antibodies Alexa Fluor 488 goat anti-mouse IgG (A11001, Thermo Scientific, Middletown, VA) and Alexa Fluor 568 goat anti-mouse IgG (A11004, Thermo Scientific, Middletown, VA) each at 1:200 dilution. Separate cells were stained with preimmune IgG instead of the primary antibody, to control for nonspecific staining. n = 20 cells per experimental group were selected. Images were captured using an Olympus DP72 digital camera (Olympus) mounted onto an Olympus BX41 microscope (Olympus). The intensity of fluorescence staining for each experimental group was calculated as follows: corrected total cell fluorescence= Integrated density – (area of cell x mean fluorescence of background readings).

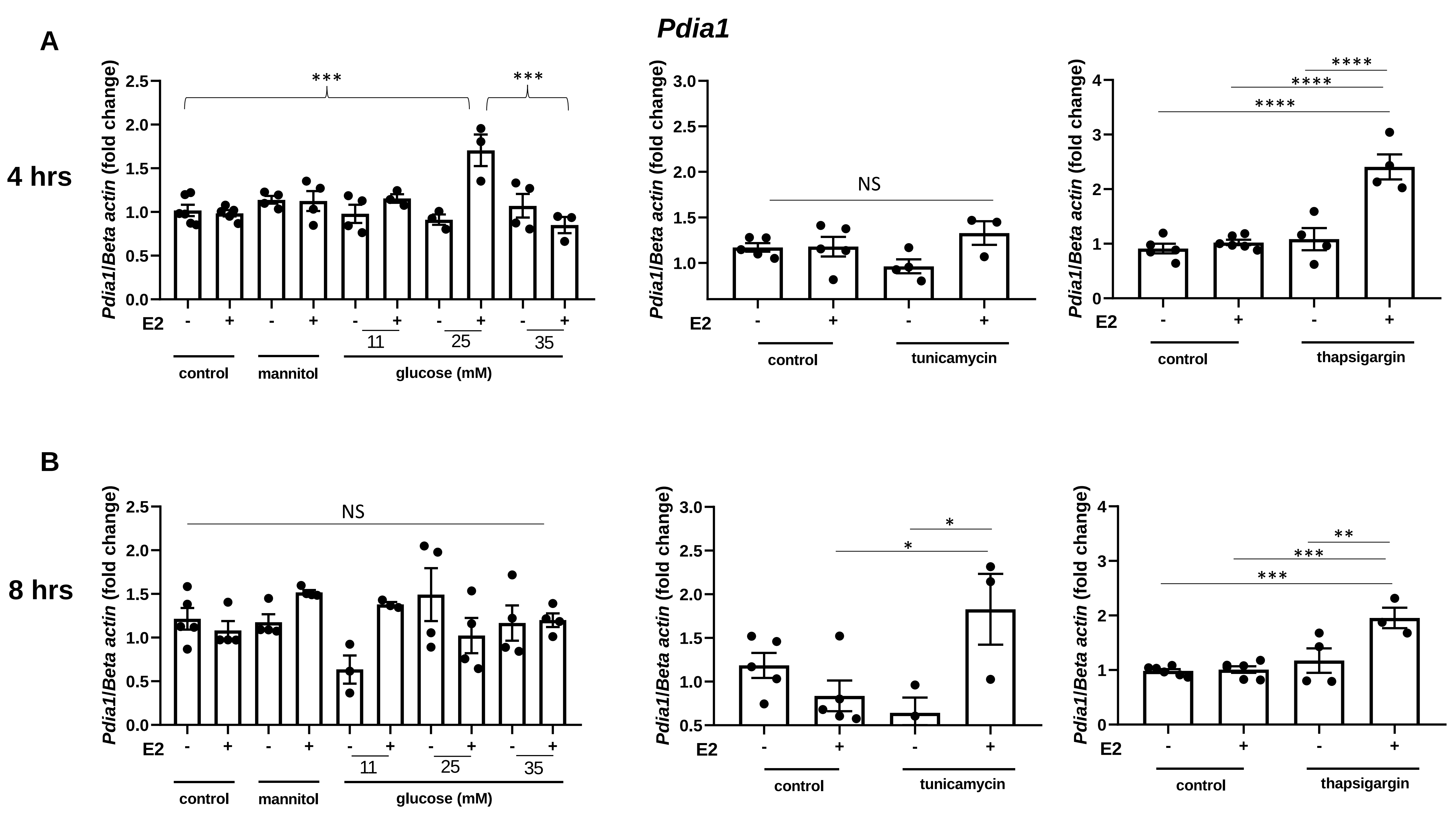
Pdia1
A
4 hrs
B
8 hrs
Pdia1/Beta actin (fold change)
E2
control
mannitol
glucose (mM)
11
25
35
tunicamycin
thapsigargin
NS
***
****
**
*

Supplementary Figure S4. Expression of *Pdia1* in BTC6 cells pretreated with estrogen. Transcripts from BTC6 cells pretreated (or not) with 17-beta estradiol (E2) were analysed for the expression of the adaptive UPR marker *Pdia1* after **(A)** 4 hours or **(B)** 8 hours of exposure to glucose (11 mM, 25 mM, 35 mM), tunicamycin (0.125 µg/ml), thapsigargin (0.25 µM). Mannitol (30 mM) was used as osmotic control. n=3-5 samples per experimental group, analyzed in duplicates. *p<0.05, **p<0.01, ***p<0.001, ****p<0.0001, NS, not significant. Bars represent standard error of the mean (SEM).

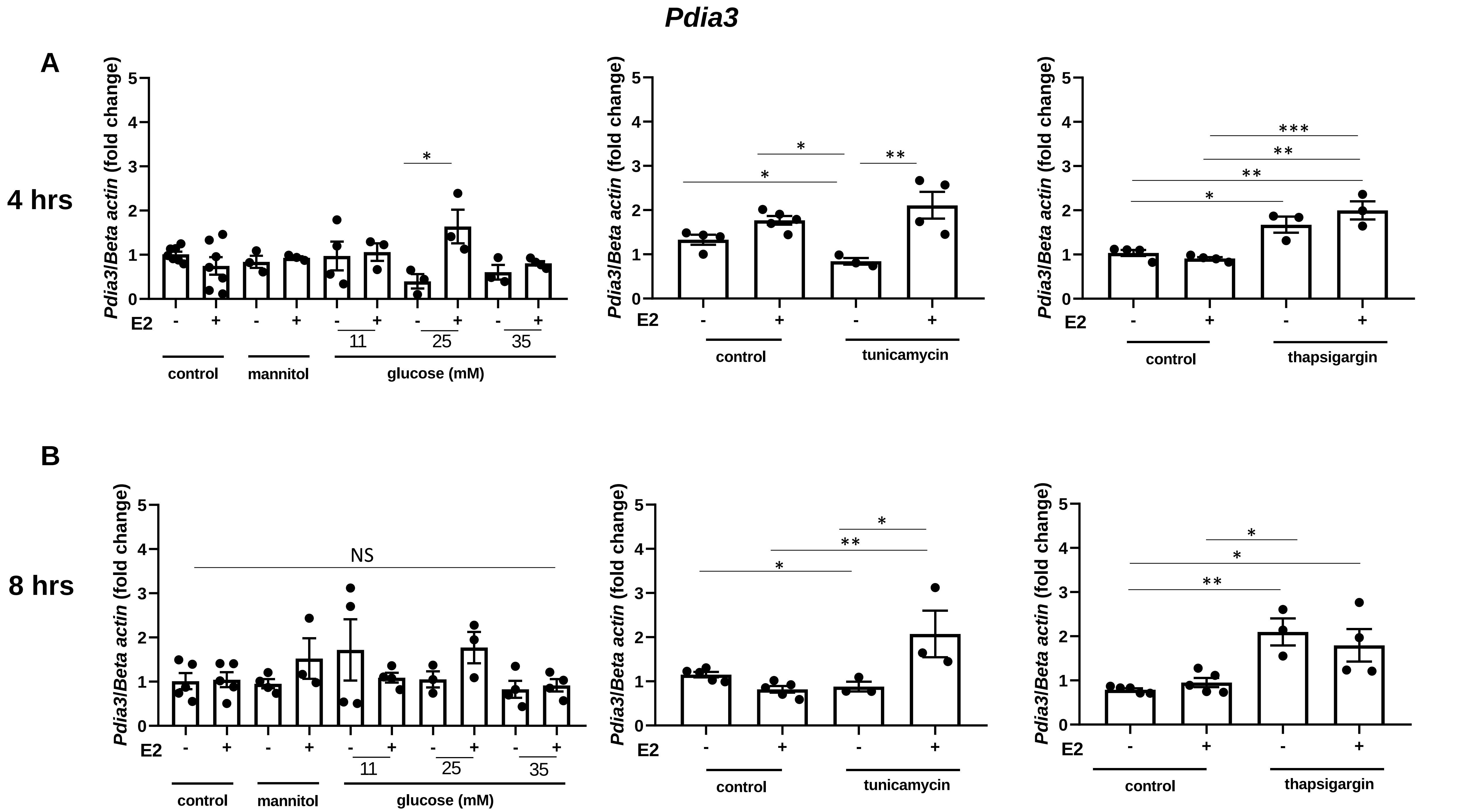

Pdia3
A
4 hrs
B
8 hrs
Pdia3/Beta actin (fold change)
E2
- +
11
25
35
control
mannitol
glucose (mM)
tunicamycin
thapsigargin
NS
*
**
***

Supplementary Figure S5. Expression of *Pdia3* in BTC6 cells pretreated with estrogen. Transcripts from BTC6 cells pretreated (or not) with 17-beta estradiol (E2) were analysed for the expression of the adaptive UPR marker *Pdia3* after **(A)** 4 hours or **(B)** 8 hours of exposure to glucose (11 mM, 25 mM, 35 mM), tunicamycin (0.125 μg/ml), thapsigargin (0.25 μM). Mannitol (30 mM) was used as osmotic control. n=3-5 samples per experimental group, analyzed in duplicates. *$p<0.05$, **$p<0.01$, ***$p<0.001$, NS, not significant. Bars represent standard error of the mean (SEM).

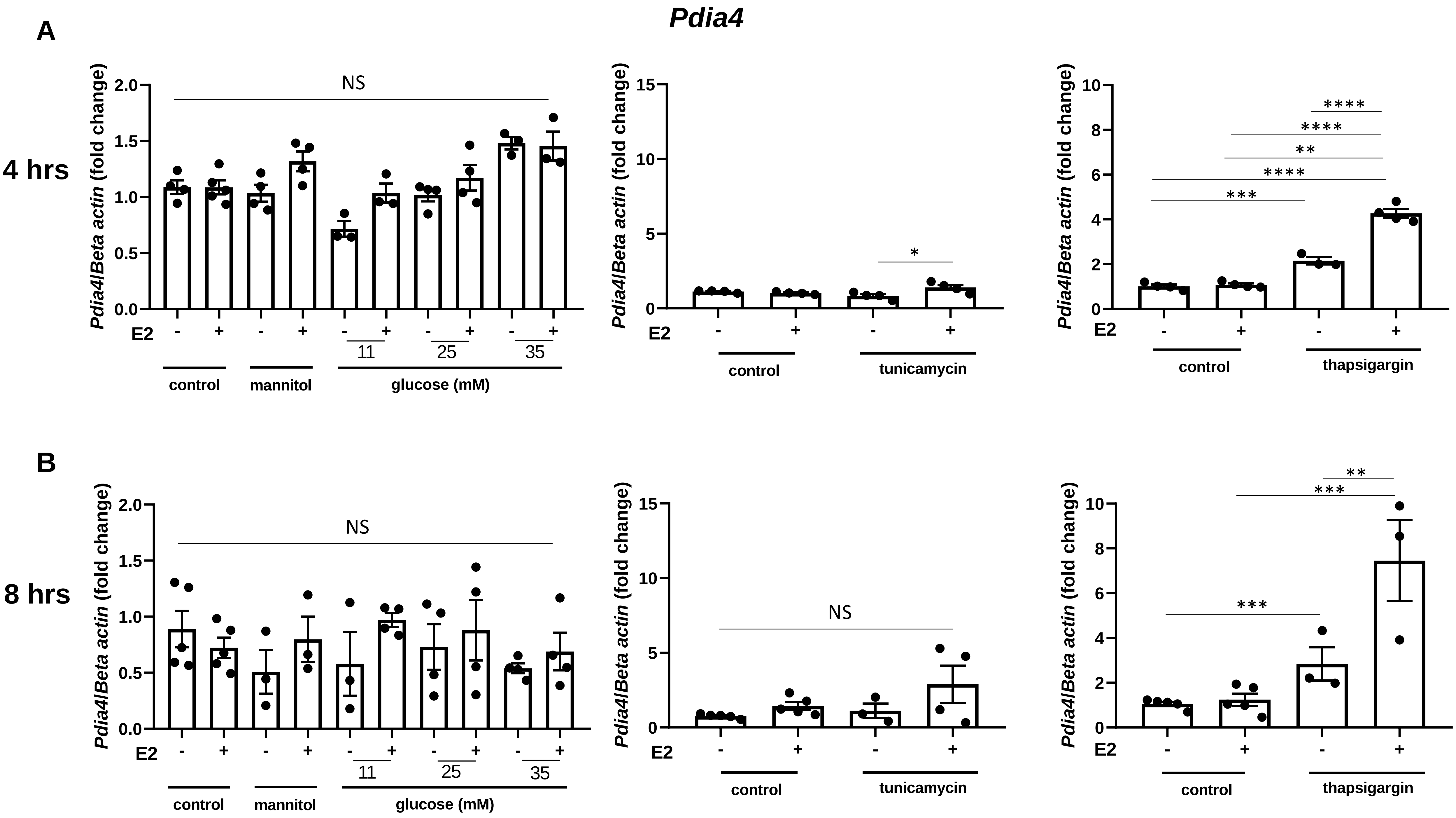

Pdia4
A
4 hrs
B
8 hrs
Pdia4/Beta actin (fold change)
NS
*
****
**
***
E2
- +
11
25
35
control
mannitol
glucose (mM)
tunicamycin
thapsigargin

Supplementary Figure S6. Expression of *Pdia4* in BTC6 cells pretreated with. Transcripts from BTC6 cells pretreated (or not) with 17-beta estradiol (E2) were analysed for the expression of the adaptive UPR marker *Pdia4* after **(A)** 4 hours or **(B)** 8 hours of exposure to glucose (11 mM, 25 mM, 35 mM), tunicamycin (0.125 μg/ml), thapsigargin (0.25 μM). Mannitol (30 mM) was used as osmotic control. n=3-5 samples per experimental group, analyzed in duplicates. *p<0.05, **p<0.01, ***p<0.001, ****p<0.0001, NS, not significant. Bars represent standard error of the mean (SEM).

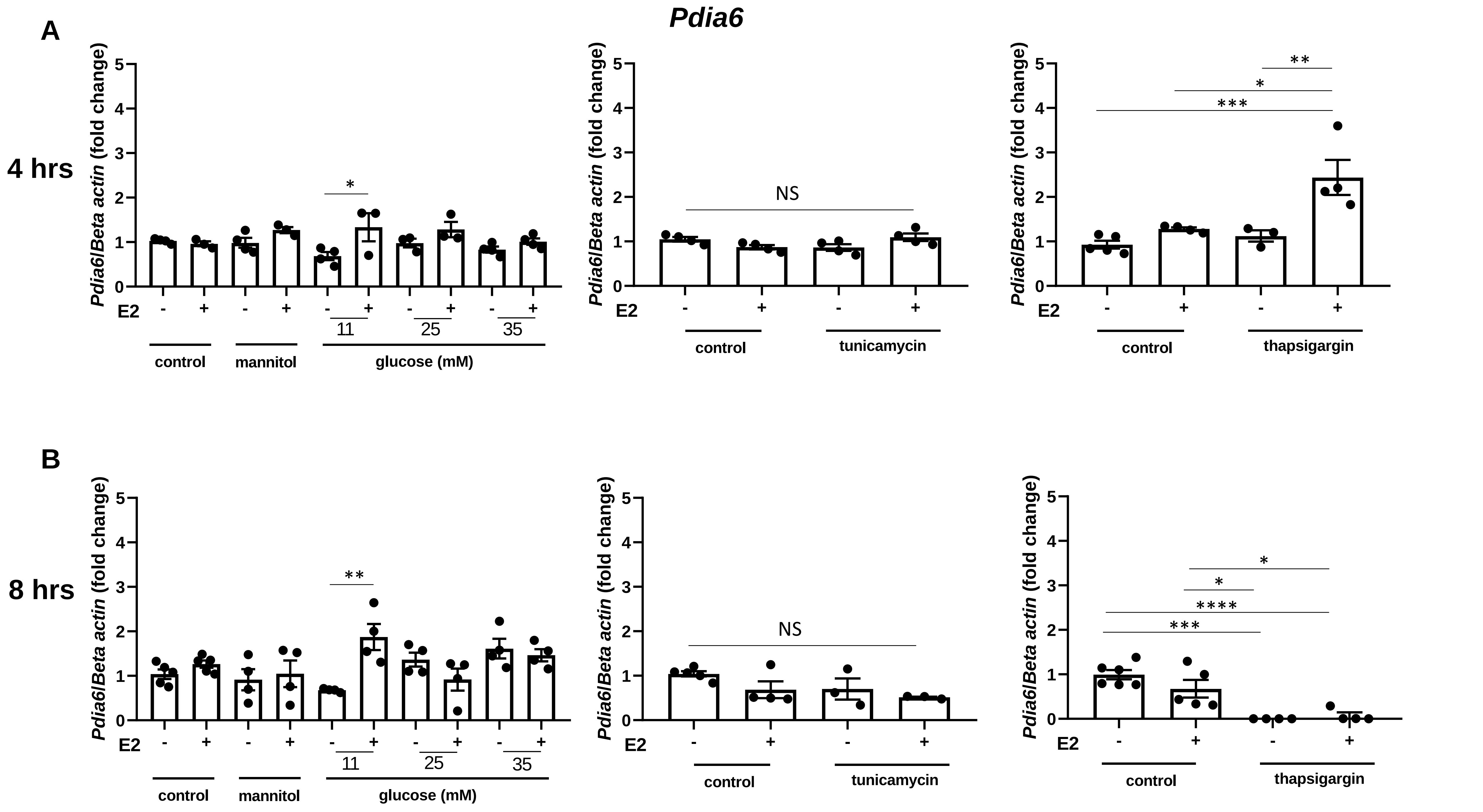
Pdia6
A
4 hrs
Pdia6/Beta actin (fold change)
E2
11
25
35
control
mannitol
glucose (mM)
NS
tunicamycin
thapsigargin
B
8 hrs

Supplementary Figure S7. Expression of *Pdia6* in BTC6 cells pretreated with estrogen. Transcripts from BTC6 cells pretreated (or not) with 17-beta estradiol (E2) were analysed for the expression of the adaptive UPR marker *Pdia6* after **(A)** 4 hours or **(B)** 8 hours of exposure to glucose (11 mM, 25 mM, 35 mM), tunicamycin (0.125 µg/ml), thapsigargin (0.25 µM). Mannitol (30 mM) was used as osmotic control. n=3-5 samples per experimental group, analyzed in duplicates. *$p<0.05$, **$p<0.01$, ***$p<0.001$, ****$p<0.0001$, NS, not significant. Bars represent standard error of the mean (SEM).

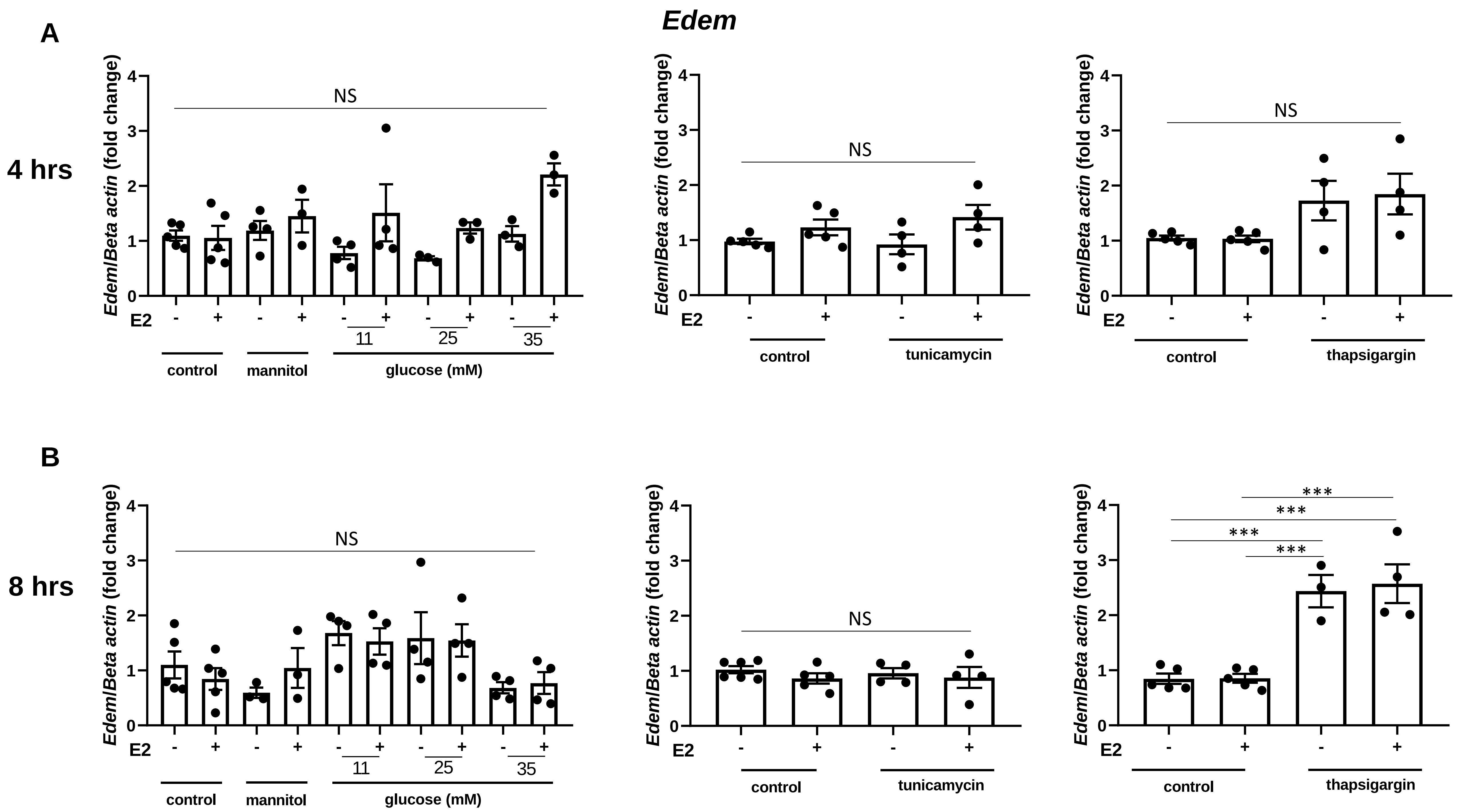

Edem
A
4 hrs
B
8 hrs
Edem/Beta actin (fold change)
NS
***
E2
- +
11
25
35
control
mannitol
glucose (mM)
tunicamycin
thapsigargin

Supplementary Figure S8. Expression of *Edem* in BTC6 cells pretreated with estrogen. Transcripts from BTC6 cells pretreated (or not) with 17-beta estradiol (E2) were analysed for the expression of the adaptive UPR marker *Edem* after **(A)** 4 hours or **(B)** 8 hours of exposure to glucose (11 mM, 25 mM, 35 mM), tunicamycin (0.125 μg/ml), thapsigargin (0.25 μM). Mannitol (30 mM) was used as osmotic control. n=3-5 samples per experimental group, analyzed in duplicates. ***p<0.001, NS, not significant. Bars represent standard error of the mean (SEM).

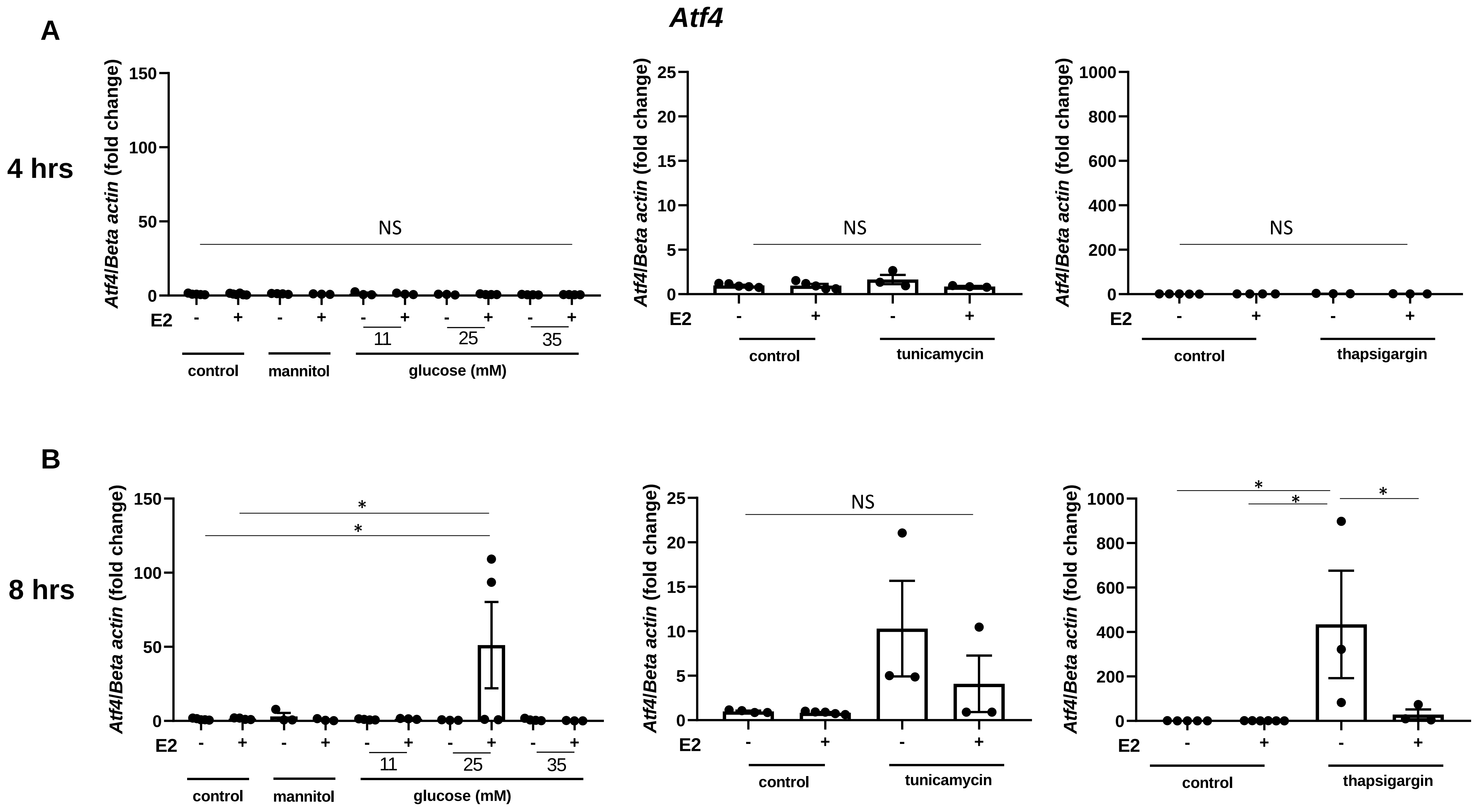
Atf4
A
4 hrs
B
8 hrs
Atf4/Beta actin (fold change)
NS
*
E2
- +
11
25
35
control
mannitol
glucose (mM)
tunicamycin
thapsigargin

Supplementary Figure S9. Expression of *Atf4* in BTC6 cells pretreated with estrogen. Transcripts from BTC6 cells pretreated (or not) with 17-beta estradiol (E2) were analysed for the expression of the adaptive UPR marker *Atf4* after **(A)** 4 hours or **(B)** 8 hours of exposure to glucose (11 mM, 25 mM, 35 mM), tunicamycin (0.125 μg/ml), thapsigargin (0.25 μM). Mannitol (30 mM) was used as osmotic control. n=3-5 samples per experimental group, analyzed in duplicates. *$p<0.05$, NS, not significant. Bars represent standard error of the mean (SEM).

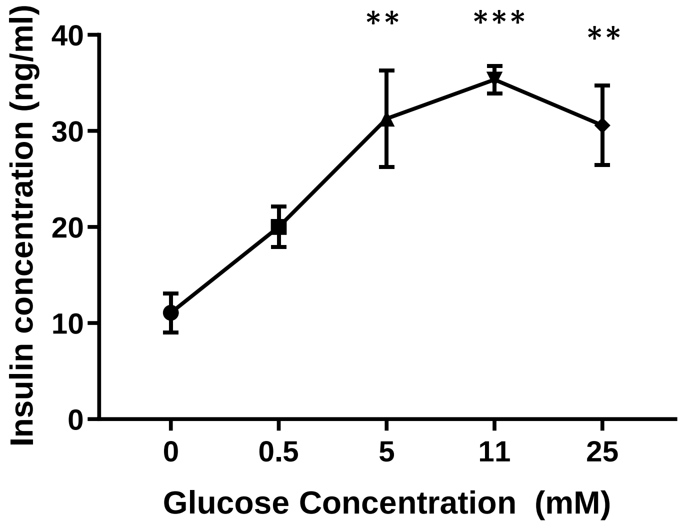

Supplementary Figure S10. Glucose-stimulated insulin secretion in BTC6 cells. BTC6 cells were exposed for two hours to Krebs-Ringer HEPES buffer containing increasing concentrations of glucose (0-25 mM). Cell medium was collected, and an enzyme-linked immunosorbent assay (ELISA) was performed to determine insulin concentration (Ultra Sensitive Mouse Insulin ELISA kit, 90080, Crystal Chem, Downers Grove, Illinois, USA). n=4 samples per experimental group, analyzed in duplicate. **p<0.01 vs 0 mM glucose; ***p<0.001 vs 0 mM glucose. Bars represent standard error of the mean (SEM).

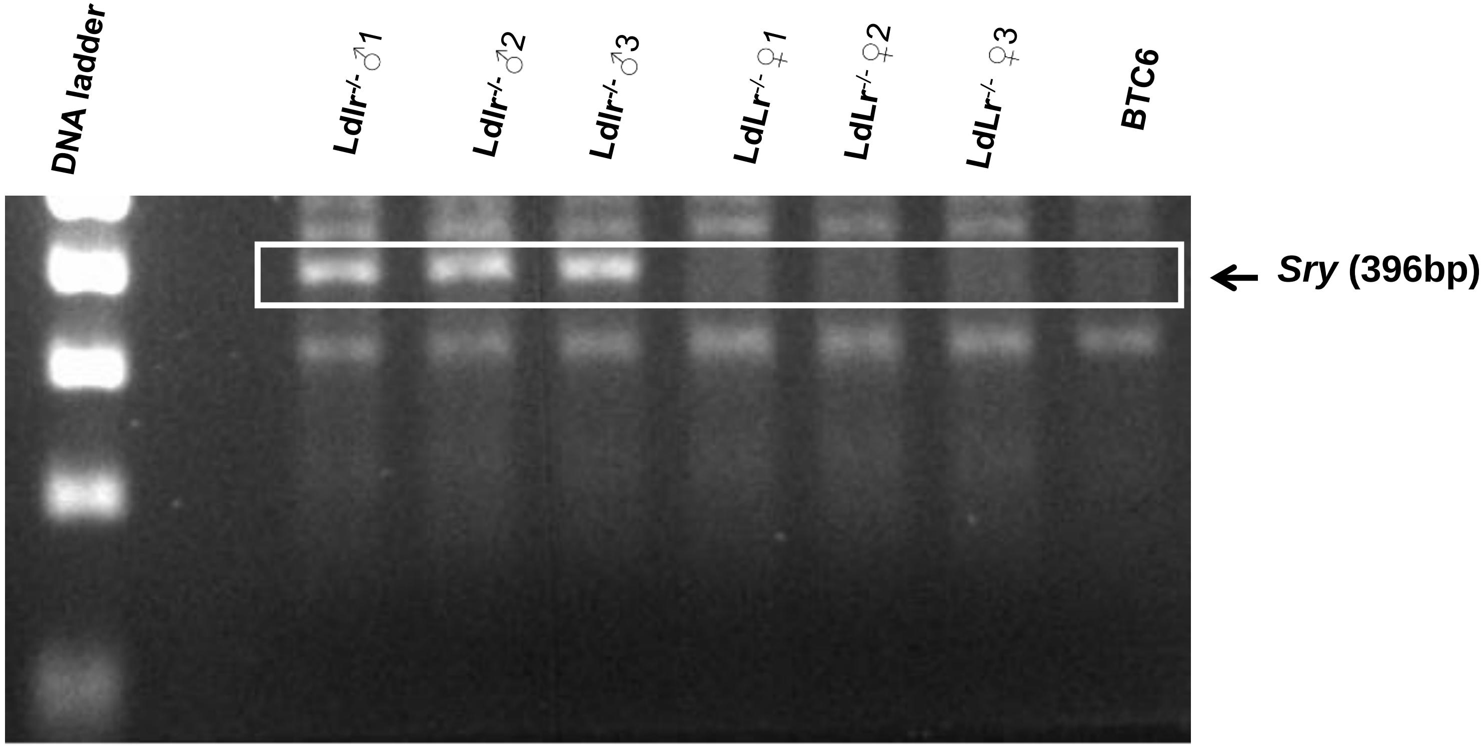

Supplementary Figure S11. Sex-identification of Ldlr$^{-/-}$ mice and mouse cell line by PCR with mouse *Sry* primers. *Sry* primers were designed based on mouse *Sry* locus, 8276-8295 and 8677 - 8658.. Mouse tails from Ldlr$^{-/-}$ mice or TC6 cell line were digested with 1 x tail lysis buffer and 2µl of DNA sample for each reaction was used.

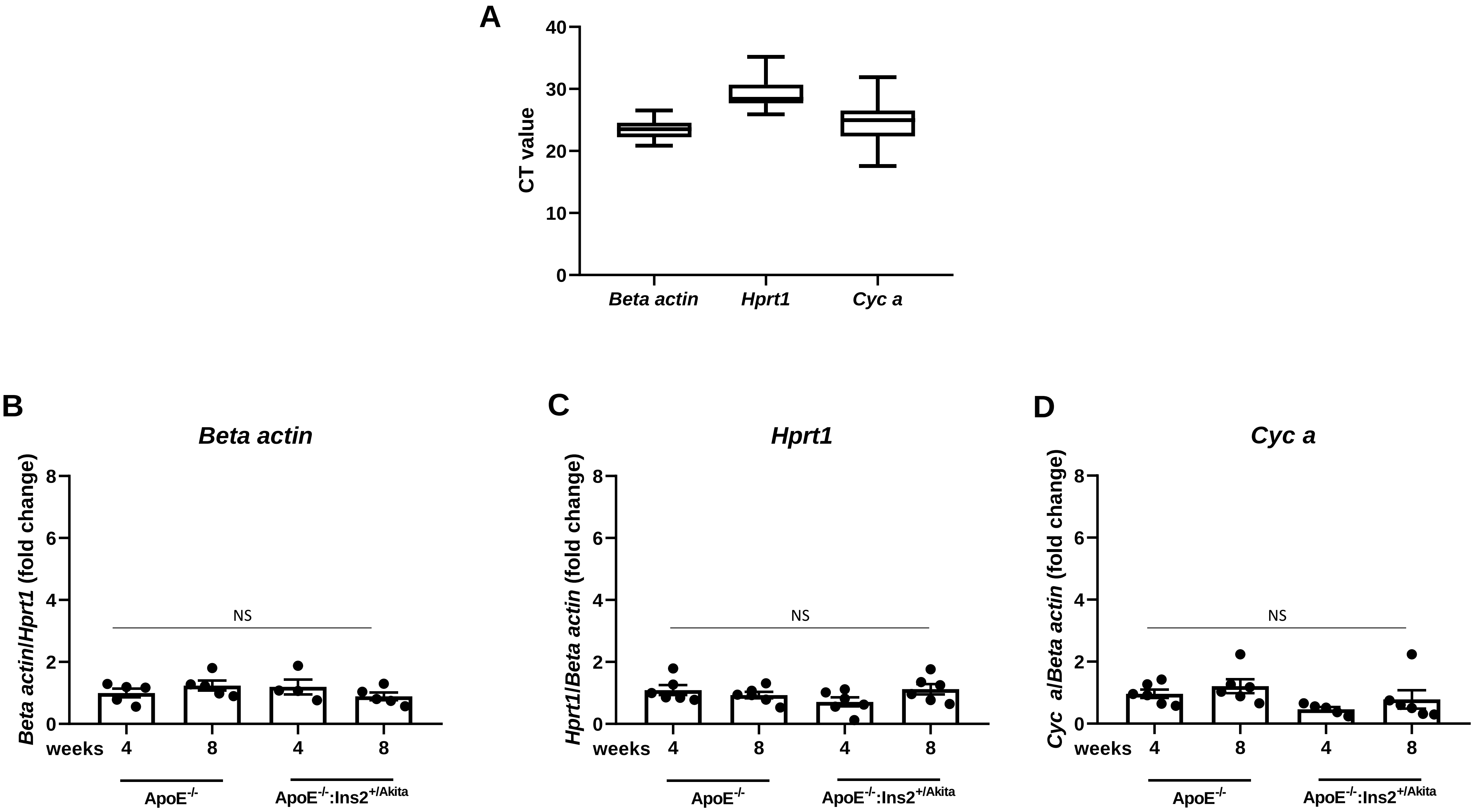
A
CT value
40
30
20
10
0
Beta actin
Hprt1
Cyc a
B
Beta actin
Beta actin/Hprt1 (fold change)
NS
weeks
4
8
4
8
ApoE-/-
ApoE-/-:Ins2+/Akita
C
Hprt1
Hprt1/Beta actin (fold change)
NS
weeks
4
8
4
8
ApoE-/-
ApoE-/-:Ins2+/Akita
D
Cyc a
Cyc a/Beta actin (fold change)
NS
weeks
4
8
4
8
ApoE-/-
ApoE-/-:Ins2+/Akita

Supplementary Figure S12. Validation of reference genes (*Beta actin*, *Cyclophilin a*, *Hprt1*) in isolated pancreatic islets. Average CT values of pooled samples of isolated pancreatic islets from male and female ApoE$^{-/-}$ and ApoE$^{-/-}$:Ins2$^{+/Akita}$ mice at 4 and 8 weeks of age for reference genes *Beta actin*, *Cyclophilin a*, and *Hprt1*. Boxes represent maximum and minimum values. Whiskers represent Standard Deviation. n=4-6 per experimental group. **(A)** Average CT values of *Beta actin*, *Cyclophilin a*, and *Hprt1* in all samples. Fold expression change of pooled samples of isolated pancreatic islets from male and female ApoE$^{-/-}$ and ApoE$^{-/-}$:Ins2$^{+/Akita}$ mice at 4 and 8 weeks of age for reference genes **(B)** *Beta actin*, **(C)** *Cyclophilin a*, and **(D)** *Hrp1*. Bars represent standard error of the mean (SEM). n=4-6 per experimental group, analyzed in duplicates.

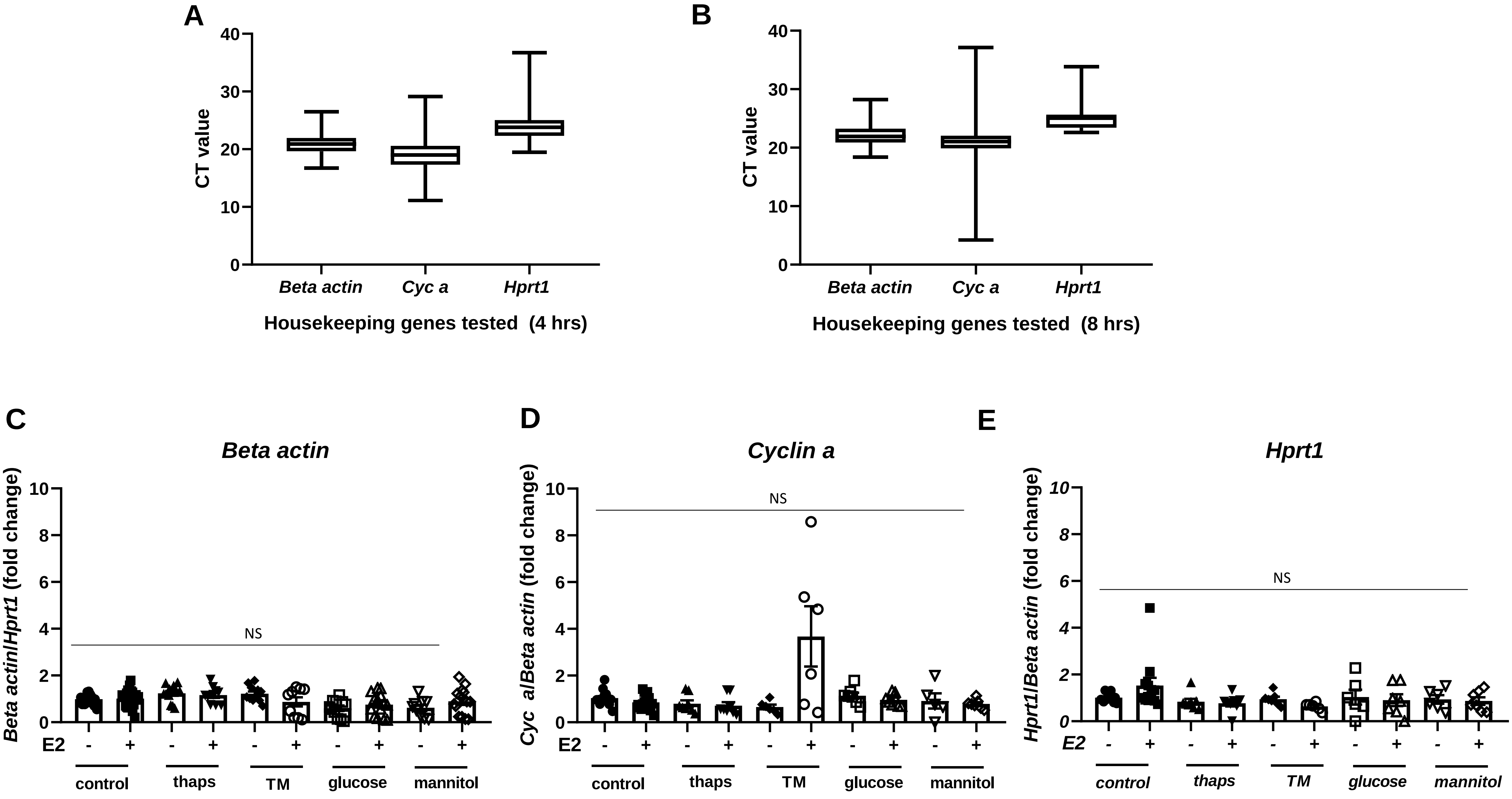
A
B
CT value
Beta actin
Cyc a
Hprt1
Housekeeping genes tested (4 hrs)
Housekeeping genes tested (8 hrs)
C
D
E
Beta actin
Cyclin a
Hprt1
Beta actin/Hprt1 (fold change)
Cyc a/Beta actin (fold change)
Hprt1/Beta actin (fold change)
NS
E2
control
thaps
TM
glucose
mannitol

Supplementary Figure S13. Validation of reference genes (*Beta actin*, *Cyclophilin a*, *Hprt1*) in BTC6 cells. Average CT values for reference genes *Beta actin*, *Cyclophilin a*, and *Hprt1* of pooled BTC6 cells pretreated (or not) with 17-beta estradiol and exposed to glucose (11 mM, 25 mM, 35 mM), mannitol (30 mM), tunicamycin (0.125 µg/ml), thapsigargin (0.25 µM) for 4 or 8 hours. Boxes represent maximum and minimum values. Whiskers represent Standard Deviation. n=8-12 per experimental group, analyzed in duplicates. **(A)** Average CT values of *Beta actin*, *Cyclophilin A*, and *Hprt1* in all samples. Fold expression change of pooled samples of pooled BTC6 cells after 4 and 8 hours exposure to glucose (11 mM, 25 mM, 35 mM), mannitol (30 mM), tunicamycin (0.125 µg/ml), thapsigargin (0.25 µM). Reference genes **(B)** *Beta actin*, **(C)** *Cyclophilin a* , and **(D)** *Hrp1* were analyzed. Bars represent standard error of the mean (SEM). n=8-12 per experimental group, analyzed in duplicates.
